# Human subjects exploit a cognitive map for credit assignment

**DOI:** 10.1073/pnas.2016884118

**Published:** 2021-01-21

**Authors:** Rani Moran, Peter Dayan, Raymond J. Dolan

**Affiliations:** ^a^Max Planck UCL Centre for Computational Psychiatry and Ageing Research, University College London, WC1B 5EH London, United Kingdom;; ^b^Wellcome Centre for Human Neuroimaging, University College London, WC1N 3BG London, United Kingdom;; ^c^Department of Computational Neuroscience, Max Planck Institute for Biological Cybernetics, 72076 Tübingen, Germany;; ^d^Department of Computer Science, University of Tübingen, 72076 Tübingen, Germany

**Keywords:** cognitive maps, reinforcement learning, decision making, model-based, model-free

## Abstract

An influential reinforcement learning framework proposes that behavior is jointly governed by model-free (MF) and model-based (MB) controllers. The former learns the values of actions directly from past encounters, and the latter exploits a cognitive map of the task to calculate these prospectively. Considerable attention has been paid to how these systems interact during choice, but how and whether knowledge of a cognitive map contributes to the way MF and MB controllers assign credit (i.e., to how they revaluate actions and states following the receipt of an outcome) remains underexplored. Here, we examine such sophisticated credit assignment using a dual-outcome bandit task. We provide evidence that knowledge of a cognitive map influences credit assignment in both MF and MB systems, mediating subtly different aspects of apparent relevance. Specifically, we show MF credit assignment is enhanced for those rewards that are related to a choice, and this contrasted with choice-unrelated rewards that reinforced subsequent choices negatively. This modulation is only possible based on knowledge of task structure. On the other hand, MB credit assignment was boosted for outcomes that impacted on differences in values between offered bandits. We consider mechanistic accounts and the normative status of these findings. We suggest the findings extend the scope and sophistication of cognitive map-based credit assignment during reinforcement learning, with implications for understanding behavioral control.

## Significance

Credit assignment (CA) to relevant actions poses a challenge because one is often flooded with reward feedback that is not easily causally attributed. We addressed this issue in a reinforcement learning framework wherein choice is mutually controlled by value-caching model-free (MF) and prospective, planning model-based (MB) systems. We find knowledge, stored in a cognitive map, filters exuberant reward feedback to guide CA in both systems but based on different attribute dimensions. In MF, CA is boosted for outcomes that are relevant (causally related) to one’s choice, whereas in MB, CA is enhanced for outcomes that attract greater attention during the deliberation process that preceded a choice. We consider normative and mechanistic accounts, including how these processes are instrumental to adaptation.

An extensive body of psychological and neuroscientific literature on dual-system reinforcement learning (RL) indicates that behavior is governed by two distinct systems (1–17)—a rigid, retrospective model-free (MF) system (18, 19) and a flexible, prospective model-based (MB) system (18, 20). Unlike an MF system, which tends to repeat actions with a past history of success, an MB system relies on a cognitive map (CM) (21), that is, a model detailing the structure of a decision-making environment, including how states, actions, observations, and rewards are linked, to predict the impact of action choice on potential future rewards. Recent research highlights competitive and cooperative interactions between these systems, including speed accuracy trade-offs (22), reliability-based arbitration (1, 23), and a plan-to-habit strategy (24), with a focus on a prospective-planning role served by the MB system during choice. Recently, we demonstrated another influence of a CM (and thus, as we described it there, MB processes) in guiding credit assignment (CA) to MF action-values (i.e., affecting how MF values of actions and states are updated as reward-outcomes are received) (25). However, by design, this influence was limited to unraveling the resolution of state uncertainty for MF purposes, leaving broader aspects of the contribution of CM-based processes to CA unexplored.

Here, we consider two potential complementary CM-based modulators of CA. Both concern the causal structure of the relationship between options and outcomes. One involves the “relatedness” of actual outcomes to an enacted choice, a retrospective effect of a CM on MF CA. The second involves the “importance” of potential outcomes during the deliberation process preceding a choice, a prospective effect of a CM on MB CA.

“Relatedness” arises out of a complexity in assigning credit when information about streams of rewards is provided that depends only partly on the actions taken (unlike situations that involve simple lotteries, for instance, when an action is directly followed by the reward it occasions). An MF system, lacking structural causal knowledge, is disposed to assign credit naively to a choice based on the entire collection of ensuing outcomes, irrespective of whether these outcomes were caused by, or related to, an actual initiating action choice. By contrast, knowledge stored as a CM can guide MF CA to favor action-related outcomes.

Take an example of a trader who deliberates purchasing one of two available mutual funds: X, which invests in companies A and B, or Y, which invests in companies A and C. Assume the trader opts for X and then later receives positive information about companies B and D. The trader might assign credit in an MF manner to her/his past action (“buy X”), updating the action’s cached value on the basis that positive consequences followed that choice. However, only one component of those positive consequences (that concerning company B) actually related to the choice of fund X. We propose that MF CA is modulated by a CM such that a change in the action’s value will be affected mostly by information about company B. More generally, relatedness depends on a causal attribution of rewards to actions (26).

We consider a second modulator of CA, termed “importance,” as a form of attentional effect. When deliberating between several choice options, and taking into account their prospective outcomes, it is often the case that certain outcomes (which we dub “unimportant”) should not determine choice, as they are common to all choice options. In contrast, other (“important”) outcomes are distinctive to some choice options but not to others, and these should be the main determinants of choice. A CM will contain this type of information and direct attention to the latter alone. We consider the possibility that when the outcomes of the choice are observed, those that garnered more attention at choice are favored in learning.

Consider our previous example where information about companies B and D triggers a CA process that leads to positive revaluation of these companies—a process useful for future MB financial decisions related to these companies. We propose this CA process can be biased by CM-based deliberations during choice. Notably, the values of companies B and C were “important” in the trader’s MB deliberation process (choosing a fund), as each is unique to one fund. The values of companies A and D, on the other hand, are less important, as these are either common to both choice options (A) or altogether absent (D). We hypothesize that representations of “important” components in a CM are activated more strongly during choice, leading to them being revalued more when information about choice outcomes are subsequently realized. Thus, ceteris paribus, the increase in the trader’s evaluation of company B will be higher than for company D, given the positive information. This evaluation is then exploited by MB planning processes for future choices.

To test these hypotheses, we developed a variant of our previously described dual-outcome bandit task (25). Participants chose between pairs of bandits (i.e., lotteries) that led to different outcomes and received a stream of reward feedback pertaining to choice-related, choice-unrelated, important, and unimportant outcomes. Critically, there are two ways to value bandits in this task. An MF controller treats each bandit holistically, and, as described above, an MB controller predicts the values of the bandits from knowledge of the outcomes to which the bandits lead as provided by a putative CM. This distinction in the structure of evaluations can then be generalized to the apportioning of credit. We consider CA to a bandit to take the form of an MF credit assignment (MFCA; since the MF system makes decisions directly based on these values). Similarly, we consider CA to the outcomes associated with the bandits to be an MB credit assignment (MBCA). To put this another way, the main distinction between MFCA and MBCA in our task is that the former pertains to a revaluation of actions, while the latter pertains to a revaluation of latent causes for these actions (i.e., the ensuing outcomes).

In support of our hypothesis that MFCA is guided by a CM, we found evidence that credit for choice-related and -unrelated outcomes is assigned to actions in a different manner. We show information about rewards actually related to chosen actions alone positively impact on the value of those actions. Information about rewards not related to chosen actions, on the other hand, have an opposite effect. Second, we found that MBCA was greater for choice outcomes that were “important” compared to “unimportant” during choice deliberations. We discuss mechanistic and normative accounts of these results.

## Results

### Behavioral Task.

**Fig. 1.**
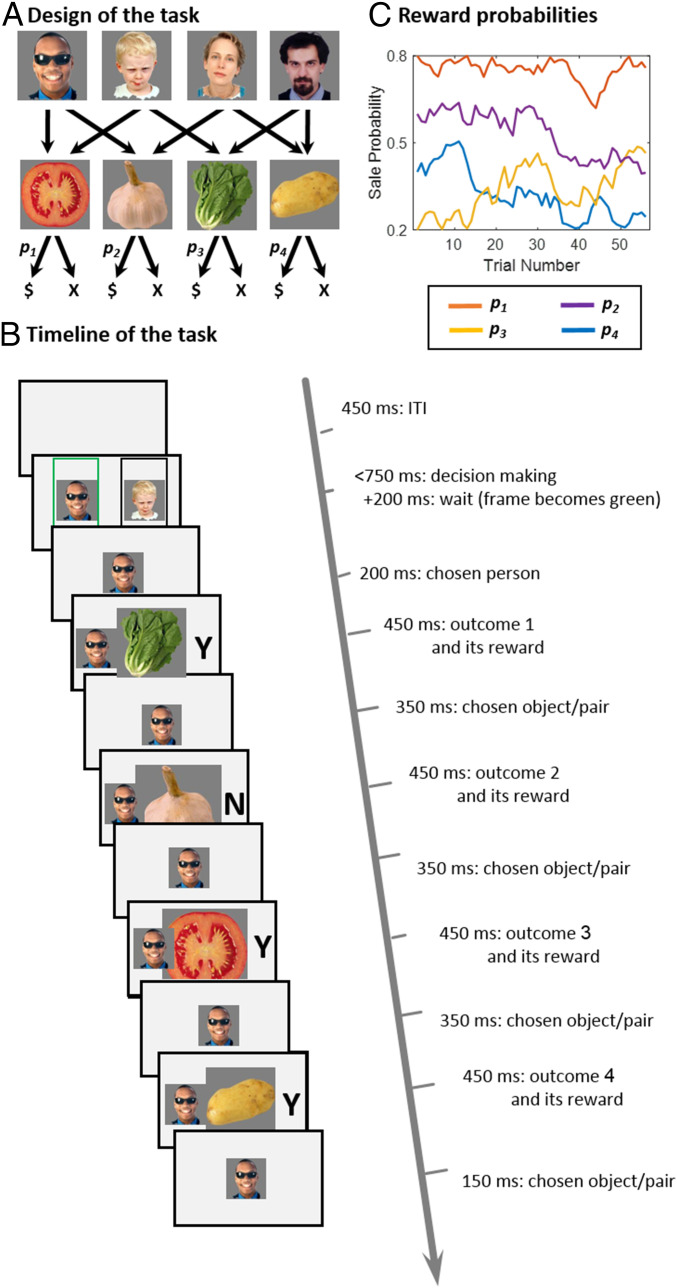
Task structure. (*A*) Participants were introduced to four people and learned which unique combination of two vegetables each grew. Each vegetable was grown by two different people. When offered in a “village market,” vegetables were sold probabilistically according to their demand. (*B*) On each trial, participants were asked to choose one of a pair of randomly offered people. At this point, participants discovered, for each vegetable, whether it sold, indicated by “Y” and “N,” respectively. In the current example, the tomato, potato, and lettuce, but not the garlic, were sold. Participant earned £1 per sale for each of the two vegetables that were grown by the person they chose. Sales of choice-unrelated vegetables did not contribute to earnings (in the illustrated trial, £1 is earned for the tomato). (*C*) One block of 56 trials is illustrated. Across trials, the market demand for the four vegetables, determining sale probability, drifted according to independent Gaussian random walks with reflecting boundaries at 0.2 and 0.8.

We designed a full-feedback variant of a dual-outcome bandit task (25). The task was introduced under a cover story. Participants were shown pictures of four different individuals and were then trained on the particular pair of vegetables each person grew in his/her garden, ensuring full knowledge of “task transition structure.” These vegetables were saleable with stochastic success in a fictional village market. By design, each person grew a unique pair of vegetables, with each individual vegetable grown by two of these four people (Fig. 1*A*).

Following training, each participant played 392 bandit trials. On each trial, subjects were given 750 ms to make a choice between a random pair of individuals who shared one vegetable as an outcome (and had one other unique vegetable outcome). Next, all four vegetables (those related to the chosen individual and those unrelated to him/her) were presented, one after the other in a random order, and participants were informed whether each vegetable had sold (Fig. 1*B*). Across the time course of the experiment, the market “demand” for different vegetables (i.e., the probabilities of being sold [rewarded]) were governed by four independently drifting random walks (Fig. 1*C*). This induced occasional changes in the ranking of the different persons in terms of expected reward probabilities (*SI Appendix*, Fig. S1), encouraging participants to learn continuously which actions are better. Participants were informed that observing all four vegetables could inform them about market demands but that their earnings on a trial would be based exclusively on the pair of vegetables grown by the person they chose. Subjects were instructed that their goal was to maximize their earnings. Participants earned a notional £1 per choice-related sold vegetable (and £0 per nonsold or choice-unrelated vegetable). Crucially, the reward-feedback stage did not provide an explicit indication about which vegetables were choice related or the notional earnings on that trial. Participants could, however, infer both of these based on exploiting an internal-task transition-structure model (i.e., a CM), which they learned during training. Henceforth, we refer to sold/unsold vegetables as rewarded/unrewarded, disregarding whether they were choice related or unrelated.

### Basic Performance Indices.

To assess whether subjects learnt the underlying vegetable/person reward probabilities, we calculated choice accuracy for each participant as the proportion of trials in which the person with the higher-expected generating-reward probability (i.e., mean of generating reward probabilities across the vegetable pair associated with that person) was chosen. The mean accuracy across participants was 0.555, significantly above a 0.5 chance-level performance (*t*(41) = 6.79, *P* = 3e-8). In comparison, the calculated accuracy rate achieved by an optimal Bayesian learner was 0.82 (*SI Appendix*, *Supplementary Methods*). To obtain a more fine-grained performance measure, which controls for trial difficulty, for each participant, we ran a logistic regression wherein the chosen display side (right = 1; left = 0) was regressed on the contrast (Right − Left) in generating reward probabilities for the two offered persons. This measure, which we term “choice sensitivity,” was positive across participants (b = 1.71, *t*(41) = 6.36; *P* = 1e-7). Additionally, choice sensitivity and accuracy strongly correlated (*r* = 0.96; *P* < 0.001). We acknowledge our task was difficult based on the across-trials average absolute difference in generating reward probabilities between pairs of offered persons (a measure of choice difficulty) varying across participants in the range 0.075 to 0.156, with a mean of 0.123. The upshot is that despite this high task difficulty, participants nevertheless assigned credit in a manner that fostered reward acquisition. We next characterize in greater detail the CA strategies participants relied on, specifically MFCA and MBCA.

### MFCA and MBCA.

We follow closely a modeling approach described in a previous study (25), which distinguishes how each putative control system (MB and MF) updates its value estimates (i.e., assigns credit) and contributes to choice performance. In brief, the MF system uses observations of rewards associated with each person from previous trials on which that person was selected to estimate a current person value (denoted $Q^{\text{MF}}$). Thus, in a trial’s choice phase, retrieved MF values ($Q^{\text{MF}}$-values) of the two people presented feed into a decision module. In a learning, reward-feedback phase, the MF system updates the $Q^{\text{MF}}$ value of the chosen person alone, a process we term MFCA. In the simplest form of MFCA (in which there is no role for a CM), an MF system lacks knowledge of state transitions, and hence, this $Q^{\text{MF}}$ value is updated to an equal extent according to information pertaining to all four vegetables, irrespective of the fact that the subject only receives rewards based on the two vegetables that the selected person grows.

A more sophisticated form of MFCA allows a CM to guide the learning process. Hence, it enacts a choice relatedness, such that reward outcomes from vegetables related to the actual choice (i.e., in this case, those grown by the chosen person) preferentially (relative to choice-unrelated vegetables) change the $Q^{\text{MF}}$ value of a choice. For example, in the trial illustrated in Fig. 1*B*, the choice-related tomato reward is predicted to reinforce the MF value of the man with sunglasses to a greater extent than each of the nonrelated lettuce and potato rewards.

By contrast, an MB system does not maintain and update values for the people directly. Instead, at choice, it calculates prospectively on-demand $Q^{\text{MB}}$ values for each person in the pair on an offer based on the arithmetic sum of the values of the two vegetables that each grows:

$$Q^{\text{MB}}\left(\text{person}\right)=Q^{\text{MB}}\left(\text{grown}\;\text{vegetable}\;1\right)+Q^{\text{MB}}\left(\text{grown}\;\text{vegetable}\;2\right).$$ [1]

During the learning phase, an MB learner updates the values of the various vegetables based on reward feedback such that the value of each vegetable increases or decreases depending on whether it sold. Henceforth, we refer to these updates as MBCA. Importantly, unlike MFCA, which does not generalize credit from one person to another, an MBCA will generalize across the two people who share a common vegetable. Thus, when a tomato is rewarded, $Q^{\text{MB}}$(tomato) increases such that during ensuing calculations, the on-demand $Q^{\text{MB}}$ values are assigned to both tomato growers as, in the example, the man with sunglasses and the child.

Another form of MBCA, which enacts our proposed importance hypothesis, does not treat all vegetables equally. For instance, in the trial illustrated in Fig. 1*B* (see also Fig. 4*A*), the vegetables fall into four categories: common to both the choice options (the tomato, grown by both people), absent from the choice options (the potato, grown by neither), exclusive to the person ultimately chosen on that trial (the garlic, grown by the man with sunglasses) alone, and counterfactual (i.e., uniquely associated with the person not chosen on that trial) (the lettuce, grown by the boy alone). Note that at choice time, the MB values of the exclusive and counterfactual vegetables affect the MB value contrast between the offered persons, as each vegetable contributes to the MB value of one of the two offered persons alone. On the other hand, the common vegetable contributes equally to the MB values of both choice options, while the absent vegetable contributes to neither value, and, hence, these vegetables do not affect the MB value contrast between the two offered individuals. Given that attention should be paid to the exclusive and counterfactual vegetables during choice, we predicted that exploitation of a CM would guide rewards for those vegetables so as to influence their $Q^{\text{MB}}$ values to a greater extent and hence impact future MB choice.

To summarize our predictions, a finding that rewards pertaining to choice-related vegetables reinforce the chosen person’s MF value to a larger extent than choice-unrelated rewards, would constitute evidence for CM guidance to MFCA. Likewise, evidence that rewards to vegetables that were important to choice reinforce MB values of these vegetables to a larger extent as compared to nonimportant vegetables would provide evidence of an “importance”-based modulation of MBCA.

In examining signatures of relatedness and importance-based influences on MFCA and MBCA to people and vegetables respectively, we first present “model agnostic” analyses, which focus on trial transitions (i.e., how trial n events affect choices on trial n+1). These analyses are supported by validating simulations using computational models, reported in *SI Appendix*, Figs. S2–S4. A full description of these models is deferred to a later section.

#### Signatures of MFCA to actions.

**Fig. 2.**
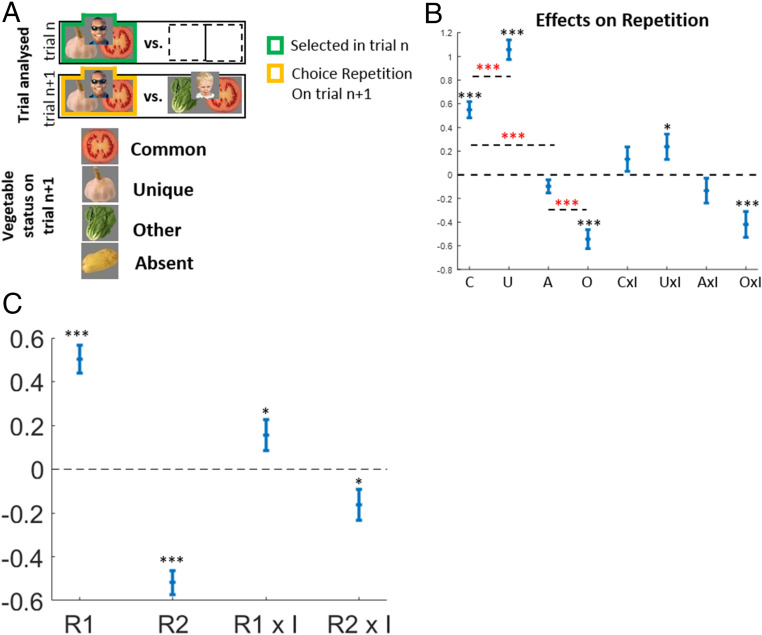
Signatures of MFCA and MBCA. (*A*) We analyzed a subset of trials that included on trial n+1 the person chosen on trial n. For clarity, we represent people by their associated pair of vegetables; in the actual experiment, participants saw images of the people alone. Places for nonchosen persons on trial n are left empty. We classified the four outcomes as Common, Unique, Other, and Absent. (*B*) Empirical effects on choice repetition. The positive Common-outcome main effect is a signature of MFCA to the chosen person based on choice-related outcomes. The positive difference between the Unique and Common effects is a signature of MBCA to choice-related outcomes. Similarly, the positive difference between the Absent and Other (labeled O) effects is a signature of MBCA to choice-unrelated outcomes. The difference between the effects of the Common and Absent outcomes is a signature of CM guidance to MFCA. The interactions between outcome-importance (labeled I) and Unique and Other outcomes are signatures that MBCA is enhanced for important, relative to nonimportant, outcomes (Fig. S2 and S4 for validating simulations). (*C*) Empirical regression effects of a trial n reward (versus nonreward) for the vegetable distinctive to the trial n+1 right-side (R1) or left-side (R2) person on right-side choices. The interaction of these rewards with the importance (denoted I) of these two vegetables on trial n is a signature of importance-based modulation of MBCA (*SI Appendix*, Fig. S3 for validating simulations). Error bars correspond to SEM across participants calculated separately in each condition (*n* = 42). *P < 0.05, ***P < 0.001. Red asterisks correspond to contrasts of effects. *P* values were calculated based on mixed-effects logistic regression models.

Anticipating our model-agnostic analysis results, we found that MFCA based on choice-related outcomes is stronger than for unrelated outcomes, supporting our hypothesis of CM guidance to MFCA. In the model-agnostic analyses, we considered only trials, n+1, which offered as a choice option of the person chosen on trial n (which we dub the “repetition person”: in Fig. 2*A*, the man with sunglasses) against another person (here, the child). We focused on these particular trials because we are interested in how trial n rewards affect choices on trial n+1. Because MFCA on a previous trial n affects the value of the trial n–chosen person alone, we cannot measure MFCA influences from trial n on trial n+1 unless the previously chosen person is offered again. Of the terms above, two are well-defined before the trial n+1 choice is made (Common: the tomato; Absent: the potato), but the other two, Exclusive and Counterfactual, are not. Since we are considering choice repetition, we define the terms Unique for the vegetable grown by the repeated person alone and not the other person (in this case, garlic), and Other for the vegetable grown by the alternative person and not the repeated person (in this case, lettuce). The Unique vegetable becomes Exclusive if a choice is repeated (the man with sunglasses) or Counterfactual if the choice is not (and the child is selected). Note also that since the trial n–chosen person is offered on trial n+1, the Common and Unique outcomes are necessarily related to the trial-n choice, whereas the Other and Absent outcome are unrelated to the trial-n choice.

We tested the degree to which choice repetition on trial n+1 depended on previous trial-n rewards for the structurally different vegetables reflecting trial n+1 categories. For example, a “Common-outcome effect” refers to the effect a trial-n reward (versus nonreward) to the common vegetable exerts on choice repetition. These reward effects provide signatures for CA processes that had occurred at the feedback phase of trial n. To test a choice-relatedness hypothesis (i.e., that MFCA [on trial n] is differentially affected by choice-related and choice-unrelated outcomes), we compared signatures of MFCA for an outcome that was either related to the trial-n choice or unrelated to that choice as follows.

##### MFCA for a choice-related outcome.

First, to assess MFCA for a trial-n choice-related outcome, we assessed the common outcome (which, as noted above, is related to the trial n choice) effect. We showed previously (25) that a positive effect of the Common outcome (in Fig. 2, the tomato) constitutes a signature of MFCA. This is because an MB system would appraise that the Common vegetable favors both trial n+1 choices and so should not influence choosing between them (*SI Appendix*, Fig. S2 and S4*A* for validating simulations). To illustrate, on trial n+1 (Fig. 2*A*), an MB system will calculate the value of the man with sunglasses as the sum of tomato and garlic values and the value of the child as the sum of tomato and lettuce values. Thus, the contrast between the MB values for these two persons equals the contrast between the garlic and lettuce values, which is invariant with respect to the tomato value. The upshot is that the value of the tomato, and particularly whether it was rewarded or unrewarded on trial n, will not affect MB deliberations on the current trial. In contrast, a trial-n Tomato reward (versus nonreward) will reinforce the MF value of the chosen person alone (man with sunglasses) and hence will affect MF choice tendencies. Thus, a common outcome-reward effect will implicate MFCA on trial n based on a common reward. Using a logistic mixed-effects model (*Methods*), in which we regressed the probability of repeating the trial-n choice on the four outcomes on trial n (and on outcome importance, as detailed below), we found (Fig. 2*B*) a main effect for the Common vegetable (b = 0.55, *t*(1333) = 7.99, and *P* = 3 × 10⁻^15^) in support of a conclusion that on trial n, credit from a choice-related vegetable was assigned in an MF manner to the person.

##### MFCA for a choice-unrelated outcome.

Second, to assess MFCA for a trial-n choice-unrelated outcome, we evaluated the effect of the Absent vegetable (which, as noted above, is unrelated to the trial n choice). Notably, a trial n reward (or lack thereof) for this vegetable will not affect MB calculations on trial n+1 (as it does not contribute to the calculated MB value of either of the offered persons). Hence, an absent reward can only exert an effect on choice via an MFCA pathway. Our logistic mixed-effects model revealed a negative absent-outcome trend (b = −0.1, *t*(1333) = −1.77, and *P* = 0.078). Thus, there is no evidence that a trial-n choice-unrelated reward reinforces positively the MF values of the chosen person. Below, we revisit this issue further in the context of our computational models. To anticipate our results, we found that credit from a vegetable unrelated to choice is assigned significantly as a negative quantity to the chosen person (i.e., decreases, rather than increases, the MF value of the chosen person).

##### A CM guides MFCA to actions.

To address our hypothesis that CM guides MFCA, we compared the effects of the common (related) and the absent (unrelated) outcomes (Fig. 2*B*). Crucially, the Common effect was larger (b = 0.65, *F*(1,1333) = 48.35 and *P* = 6 × 10^−12^), in support of our hypothesis. Indeed, in the absence of CM-based guidance, MFCA should be observed to an equal extent for all outcomes (*SI Appendix*, Figs. S2 and S4*C* for validating simulation).

#### Signatures of MBCA to outcomes.

Prior to testing our second hypothesis pertaining to importance-based MBCA, we show that MBCA occurs for both choice-related and -unrelated vegetables.

##### MBCA for a choice-related outcome.

To assess MBCA to a trial-n choice-related outcome, we compared the Unique and the Common reward effects on choice repetition. Note that both the common and unique vegetables are related to the trial n choice. However, unlike a common reward that fosters choice repetition via an MF pathway alone (for reasons explained above), a unique reward contributes to choice repetition via both the MF pathway (reinforcing the trial n-chosen person, hence increasing the tendency to repeat its choice) and the MB pathway (reinforcing of the unique vegetable that can be obtained on trial n+1 only by repeating the choice). Our logistic mixed-effects model revealed that a choice-repetition effect of reward versus nonreward on trial n was stronger for the Unique than the Common vegetable (b = 0.51, *F*(1,1333) = 43.6, and *P* = 6 × 10^−11^; Fig. 2*B*). This finding supports a conclusi on that MBCA occurs on trial n for choice-related outcomes (*SI Appendix*, Figs. S2 and S4*B* for validating simulations).

##### MBCA for a choice-unrelated outcome.

Considering MBCA to trial-n choice-unrelated outcomes, we found the Other vegetable effect was more negative than an Absent vegetable effect (b = −0.45, *F*(1,1333) = 30.74, *P* = 4 × 10^−8^; Fig. 2*B*). This finding supports a conclusion that on trial n, an MBCA occurs for outcomes unrelated to choice (note that both the Absent and Other vegetables are unrelated to the trial n choice). Indeed, while both outcomes influence choice repetition similarly via an MF pathway, Other, but not Absent, rewards (versus nonrewards) on trial n contribute to reducing choice repetition via an MB pathway since by not repeating the choice, one obtains the Other outcome (*SI Appendix*, Figs. S2*D* and S4*B* for validating simulation). In sum, these results support a conclusion that MBCA occurs for both (trial n) choice-related and choice-unrelated outcomes. Additionally, the difference between these two contrast effects, for the related and unrelated outcomes, was nonsignificant (b = 0.06, *F*(1,1333) = 0.36, and *P* = 0.55), providing no support for the idea that the extent of MBCA to outcomes differs as a function of choice relatedness.

#### MBCA is enhanced for important outcomes.

Next, we tested our second hypothesis, namely, that MBCA was enhanced for outcomes that were “important” during MB choice deliberations. We operationalized this prediction as Unique and Other outcome effects on choice repetition being stronger when these outcomes were “important” on trial n compared to being “unimportant.” For example, this implies that the MB value of garlic (Unique) will increase to a greater extent after receiving a reward on trial n if garlic was “important” versus “unimportant” on that trial. This would then induce a stronger MB effect on choice repetition on trial n+1. Thus, our hypothesis predicts a positive interaction between the Unique outcome and importance on choice repetition and, for similar reasons, a negative Other outcome × importance interaction.

To test these predictions, our logistic mixed-effects model included an “Importance” indicator that captures whether the (trial n+1) Unique and Other vegetables were “important” on trial n. We found (Fig. 2*B*) a positive interaction effect between “Importance” and the Unique outcome (b = 0.24, *t*(1333) = 2.22, and *P* = 0.027). A simple effects analysis revealed that the Unique outcome effect was positive when it was “unimportant” (b = 0.94, *F*(1,1333) = 80.56, and *P* = 9 × 10^−19^) but even more so when it was “important” (b = 1.17, *F*(1,1333) = 162.51, and *P* = 3 × 10^−35^) on trial n. Similarly, we found a negative interaction effect between Importance and the Other outcome (b = −42, *t*(1333) = −3.85, and *P* = 1 × 10^−4^) such that the simple Other outcome effect was negative when it was “unimportant” (b = −0.33, *F*(1,1333) = 14.91, and *P* = 1 × 10^−4^) but more so when it was “important” (b = −0.75, *F*(1,1333) = 50.42, and *P* = 2 × 10^−12^) on trial n. These findings support the hypothesis that MBCA is enhanced for “important” versus “unimportant” outcomes (*SI Appendix*, Figs. S2 and S4*D* for validating simulation). Note, the main effects for both Common and Absent outcomes reported earlier were not qualified by interactions with “Importance” (both *P* > 0.2; Fig. 2*B*). Thus, there is no evidence that MFCA is modulated by “importance” (note that if the Unique and Other vegetables are important, then the Common and Absent vegetables are unimportant and vice versa).

#### Further evidence that importance modulates MBCA.

Given the nature of learning, the strongest effects of outcomes on a trial are expected to be evident on the very next trial. In order to probe MFCA, we have so far concentrated our model-agnostic analyses on the half of the trial-to-trial transitions for which trial n+1 featured the trial n choice. However, no such restriction is necessary to probe MBCA since outcomes on trial n affect choices on trial n+1 even when the person chosen on trial n is not on offer. To address this limitation, we performed an additional analysis that provided a signature of enhanced MBCA for important outcomes, one based on all trial transitions (Fig. 2*C*). By design, on each trial (n+1), the two offered individuals grow one vegetable in common, and each grows one unique vegetable (to avoid confusion, here we call these two vegetables distinctive). Rewards for these (trial n+1) distinctive vegetables administered on trial n will increase the tendency to choose the corresponding persons on trial n+1 via an MB pathway. If, as hypothesized, MBCA is enhanced for important outcomes, then these reward effects should be stronger when the two distinctive vegetables were important versus unimportant on trial n.

To test this prediction, we used a mixed-effects logistic model (*Methods*) to regress the display side of the chosen person (right = 1; left = 0) on whether his/her distinctive vegetable was previously (trial n) rewarded (denoted R1), whether the other (left side) person’s distinctive vegetable was previously rewarded (denoted R2), and whether these two vegetables were important on the previous trials (denoted I). We found that a previous reward to each person’s distinctive vegetable increased the probability to choose that person (Right: b = 0.5, *t*(134) = 7.93, and *P* = 4 × 10^−14^; Left: b = −0.52, *t*(134) = −9.67, and *P* = 2 × 10^−19^) and that critically, these effects were stronger when the two distinctive vegetables were important on the previous trial, as evident from the interactions between rewards and importance (Right: b = 0.16, *t*(134) = 2.15, and *P* = 0.032; Left: b = −0.16, *t*(134) = −2.35, and *P* = 0.02). Notably, whereas MFCA influences could account for the reward effects, influences of MBCA alone account for the interactions with importance (*SI Appendix*, Figs. S3 and S4*E* for validating simulations). These findings converge with those reported in the previous section and again support our hypothesis that MBCA is enhanced for important outcomes.

### Computational Modeling.

The behavioral analyses we report above are limited in so far as they consider the effects on adjacent trials alone, whereas the actions of RL agents are subject to influences from an entire task history. To address this limitation, we tested a series of computational models that were fitted to the entire dataset and specified the likelihood of choices based on the entire choice history. Furthermore, computational modeling allowed us to characterize MBCA and MFCA processes with a finer resolution by probing potential interactions between choice relevance and importance.

We modeled choices as arising from a mixture of MB and MF contributions. In our full model, each outcome (Common, Absent, Exclusive, and Counterfactual, see Fig. 4*A*) was endowed with two CA parameters that contributed to updates of MF person values and MB vegetable values (*Methods*) and were free to vary. For example, the MFCA Absent parameter prescribes the extent to which the MF value of a chosen person increases/decreases during the feedback stage, when the absent vegetable is sold/not sold. Similarly, the MBCA Counterfactual parameter controls the extent to which the MB value of the counterfactual vegetable increases/decreases during the feedback stage when this vegetable is sold/not sold. Critically, this model supports good parameter recovery (*SI Appendix*, *Supplementary Methods* and Figs. S5 and S6), allowing us to probe estimated CA parameters to test our hypotheses. Note, the Common and Exclusive parameters pertain to choice-related outcomes. Similarly, the Exclusive and Counterfactual parameters pertain to important outcomes as the values of these outcomes contribute to a contrast between MB values of the two offered persons during choice.

First, to validate use of our model, we conducted ablation studies, creating a set of five submodels by knocking out different cognitive aspects of CA processes in the full model, allowing us to test the following set of hypotheses that each component does not contribute to performance in our task: 1) To test the hypothesis of no MFCA contribution in our task, we formulated a “pure MB” submodel, which set all four MFCA parameters to 0; behaviorally, this model should not predict our model-agnostic signatures pertaining to MFCA. 2) Similarly, to test the hypothesis of no MBCA contribution to performance, a “pure MF” submodel was formulated, which set all four MBCA parameters to 0; behaviorally, this model should not predict our model-agnostic signatures pertaining to MBCA. 3) To test the hypothesis that MFCA may affect performance without guidance from a CM, we formulated a “no CM guidance for MFCA” submodel. This forced all four MFCA parameters to be equal; behaviorally, this model should not predict model-agnostic signatures in which there is enhanced MFCA for related as compared to unrelated outcomes. 4) To test the hypothesis that unrelated rewards do not affect MFCA, a “no MFCA for unrelated choice outcomes” submodel was formulated, which constrained the MFCA parameters for the choice-unrelated (i.e., Absent and Counterfactual) outcomes to 0; behaviorally, this model should not predict a model-agnostic signature pertaining to negative MFCA for unrelated outcomes (note models 3 to 4 included the four MBCA parameters). 5) To test the hypothesis that MBCA is not modulated by outcome importance, we formulated an “egalitarian MBCA” submodel, which constrained all four MBCA parameters to be equal (note this model still had the four-parameter MFCA component); behaviorally, this model should not predict our model-agnostic signatures pertaining to importance-based modulation of MBCA.

We compared each of these submodels in turn to our full model using a bootstrap generalized-likelihood ratio test (25, 27) (*SI Appendix*, *Supplementary Methods*). In brief, this method is based on classical statistical hypothesis testing, where for model comparisons, a sub model serves as the H0 null hypothesis and the full model as the alternative H1 hypothesis. We verified also that our model comparison method was suitably powered to reject each of the submodels at the group level (*SI Appendix*, *Supplementary Methods*). Below, we report for each model both the number of participants for which it was rejected (note our task was not designed to have high power for individual participants), as well as a “group-level” test, for rejecting the null hypothesis that all participants rely on the submodel. Note rejecting the submodel at the group level supports an inference that a subset of participants rely on the submodel, but it does not imply the full model is more prevalent in the subject population (we used a different approach for assessing the prevalence of the various submodels—see below).

**Fig. 3.**
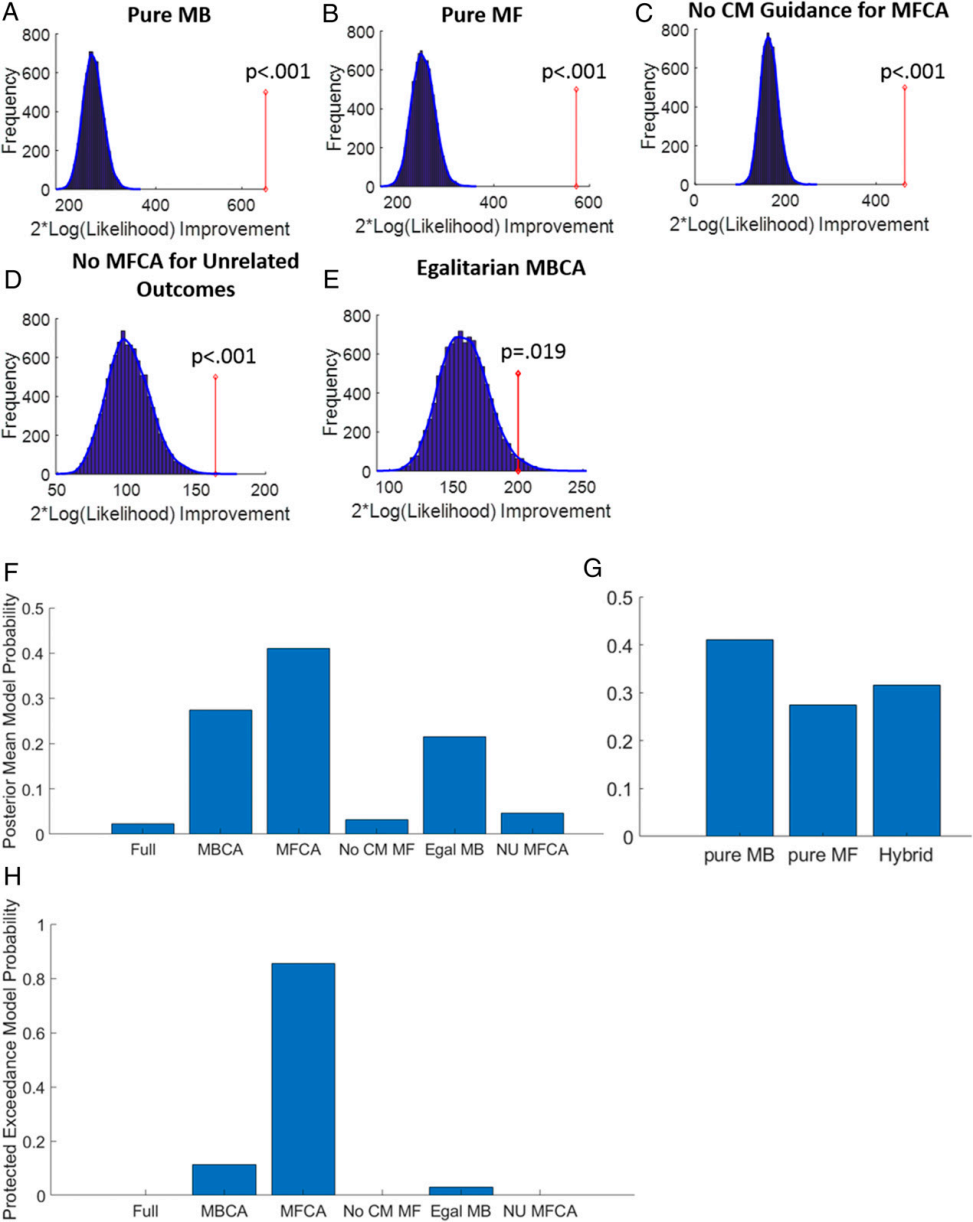
Model-comparison results. (*A*) Results of the bootstrap-GLRT model comparison for the pure MBCA submodel. The blue bars show the histogram of the group twice log-likelihood improvement (model versus submodel) for synthetic data simulated using the submodel (10,000 simulations). The blue line displays the smoothed null distribution (using MATLAB’s “ksdensity”). The red line shows the empirical group twice log-likelihood improvement. A total of 0 out of the 10,000 simulations yielded an improvement in likelihood that was at least as large as the empirical improvement. Thus, the submodel can be rejected with *P* < 0.001. (*B*–*E*) Same as *A* but for the pure MFCA (*P* < 0.001), the no CM guidance for MFCA (*P* < 0.001), the no MFCA for choice-unrelated outcome (*P* < 0.001), and the egalitarian MBCA (*P* = 0.019) submodels. (*F*) The posterior expected probabilities of the different models in the population, obtained using on hierarchical Bayesian model selection method. “Full”: Full model. MBCA/MFCA: pure MBCA. MFCA submodels: No CM. MF: no CM guidance for MFCA submodel. Egal MB: Egalitarian MBCA submodel. NU MFCA: No MFCA for unrelated-choice outcomes submodel. (*G*) Posterior expected model probabilities in the population when pooling together the various Hybrid models (Full; NO CM MF; Egal MB; and NU MFCA). (*H*) Protected exceedance probabilities for the various models.

First, we rejected both the pure MB (group level *P* < 0.001; 23 individuals, *P* < 0.05; Fig. 3*A*) and the pure MF (group level: *P* < 0.001; 13 individuals; Fig. 3*B*) submodels. These results are consistent with both MBCA and MFCA processes contributing to choice in our task. Second, we rejected the “no CM guidance for MFCA” submodel (group *P* < 0.001; 20 individuals; Fig. 3*C*), supporting our hypothesis that a CM guides MFCA (below, we show MFCA was higher for related- than unrelated-choice outcomes). Third, we rejected the “no MFCA for unrelated-choice outcomes” submodel (group, *P* < 0.001; eight individuals; Fig. 3*D*), supporting a conclusion that MFCA occurs for unrelated vegetables (below, we show that this CA is negative). Finally, we rejected the “egalitarian MBCA” submodel (group, *P* = 0.021; three individuals; Fig. 3*E*), supporting the conclusion that outcome differs in their extent of MBCA (below, we show that MBCA is boosted for important outcomes). Additionally, we verified that unlike the full model, which predicted all the model-agnostic signatures presented above, each of its submodels failed to predict a subset of these effects (*SI Appendix*, Figs. S2–S4).

#### Assessing model prevalence.

While the above model comparisons allowed us to reject each submodel in favor of the full model for a subset of participants, they do not address a question pertaining to the prevalence of different models in the population. For the latter question, we used a robust hierarchical Bayesian model selection approach, which takes account of interindividual heterogeneity, treating models as random effects that could differ across subjects (28, 29) (*SI Appendix*, *Supplementary Methods*). Fig. 3*F* presents the posterior expected probability of each model in the population (these are normalized concentration parameters from the posterior Dricihlet distribution; *SI Appendix*, *Supplementary Methods*). Pooling together the various hybrid models, which include contributions from both MBCA and MFCA, revealed about 41% of the population is expected to rely on pure MFCA processing, another 27% rely on pure MBCA processing, and the remaining 32% is expected to exploit a form of Hybrid control (Fig. 3*G*). We also calculated protected exceedance probabilities, that is, the probabilities that each model is the most prevalent model in the population (taking into account that apparent difference in model-frequencies may be due to “chance”; Fig. 3*H*). The protected exceedance probabilities for the pure MFCA, pure MBCA, and egalitarian MBCA were 0.86, 0.11, and 0.03, respectively, and nearly zero for the other models.

##### Analyses based on CA model parameters.

**Fig. 4.**
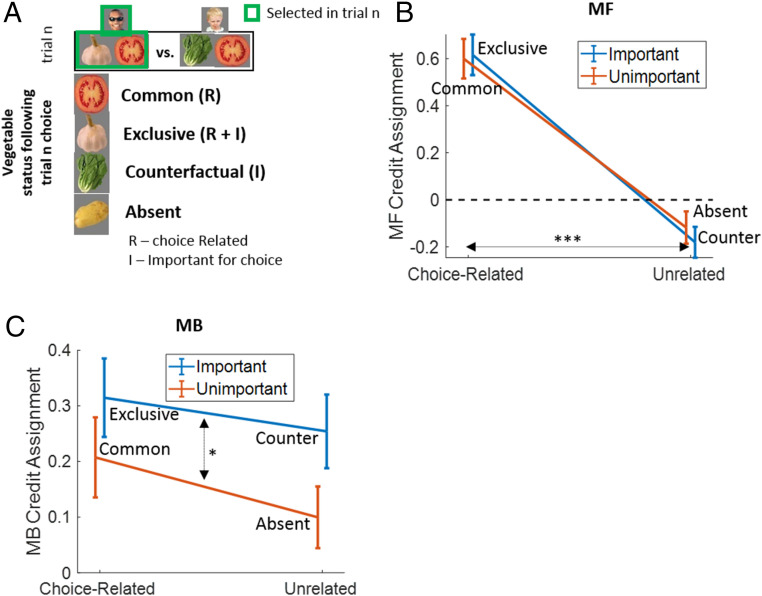
Signatures of MF and MB CA. (*A*) During a post-choice reward-feedback stage, outcomes were designated as Common, Exclusive, Counterfactual, and Absent. Two outcomes were choice-related (R) and two are were “important” (I) with respect to MB deliberations. (*B*) MFCA parameters based on the full model. The choice-relatedness effects demonstrate CM-based guidance of MFCA. Furthermore, the mean negativity for choice-unrelated outcomes implicates negative MFCA for choice-unrelated outcomes. (*C*) Similar to B but for the MB system. The “importance” main effects show that MBCA is enhanced for important outcomes. Error bars correspond to SEM across participants calculated separately in each condition (*n* = 42). *P < 0.05, ***P < 0.001. *P* values were calculated based on mixed-effects logistic regression models.

We next probed whether MBCA and MFCA parameters varied as a function of whether an outcome on a trial was “important” (Exclusive and Counterfactual) or “unimportant” (Common and Absent) for MB choice deliberations and whether it was related (Common and Exclusive) or unrelated (Counterfactual and Absent) to the choice (Fig. 4*A*). The following analyses rely on maximal likelihood (ML) parameters for the full model, an approach justified by the fact that each of our submodels can be viewed as an instance of the full model. For example, if a participant is characterized by the pure MBCA submodel, then at the same time, he/she is characterized by the full model with MFCA equal to 0. Hence, the full model comprises a unifying measurement tool for assessing CA patterns, disregarding model heterogeneity between participants. We note, however, the prevalence of the full model in the population was estimated to be very low (Fig. 3*F*). Thus, we supplement our analysis using a Bayesian averaging (BA) approach that takes into account heterogeneity across participants in generating models.

In our first analysis, we tested our hypothesis pertaining to CM guidance to MFCA, regressing, using a mixed-effects model, the four ML MFCA full-model parameters for each participant (4 × 42 parameters in total) on indicators specifying whether a parameter pertained to importance (I) and choice-relatedness (R) outcomes (*SI Appendix*, *Supplementary Methods*). We found a positive main effect for choice “relatedness” (b = 0.76, *t*(164) = 7.16, and *P* = 3 × 10^−11^). Neither the main effect for “importance” (b = −0.02, *t*(164) = −0.41, and *P* = 0.686) nor the interaction between “importance” and “relatedness” (b = 0.08, *t*(164) = 0.7, and *P* = 0.484) were significant (Fig. 4*B*). These results support our conclusion that a CM guides MFCA such that rewards for related outcomes reinforce MF values of a chosen bandit more than unrelated rewards. Strikingly, whereas MFCA for choice-related vegetable was positive (b = 0.61, *F*(1,164) = 65.88, and *P* = 1 × 10^−13^), it was negative for choice-unrelated vegetables (b = −0.15, *F*(1,164) = 8.43, and *P* = 0.004). Thus, not only do choice-unrelated outcomes not provide positive reinforcement, but they actually reinforce choices negatively.

A similar mixed-effects model for the MB system (*SI Appendix*, *Supplementary Methods*), used to test our hypothesis pertaining to importance modulation on MBCA, revealed a positive main effect for “importance” (b = 0.13, *t*(164) = 2.48, and *P* = 0.014). Neither the main effect for choice “relatedness” (b = 0.08, *t*(164) = 1.28, and *P* = 0.204) nor the interaction between “importance” and “relatedness” (b = −0.05, *t*(164) = −0.65, and *P* = 0.519) were significant (Fig. 4*C*). Furthermore, MBCA was positive for both “important” (b = 0.28, *F*(1,164) = 25.04, and *P* = 1 × 10^−6^) and “unimportant” (b = 0.15, *F*(1,164) = 9.38, and *P* = 0.003) outcomes. These results support our hypothesis that choice deliberations based on a CM bias MBCA. Indeed, while MBCA occurs for both important and unimportant outcomes, it is enhanced for the latter. Furthermore, when we calculated a separate importance-based modulation effect on MBCA for related (contrast: Exclusive − Common MBCA parameters) and unrelated outcomes (contrast: Counterfactual − Absent MBCA parameters), we found a positive correlation between the two (*r* = 0.38, *P* = 0.012; *SI Appendix*, Fig. S10). Thus, importance-based modulation of MBCA is coordinated across related and unrelated outcomes. Additionally, we verified that the interpretation of our two effects pertaining to outcome relatedness and importance are not confounded by parameter trade-offs (*SI Appendix*, *Supplementary Methods* and Figs. S7–S9), and we found no evidence that either MFCA or MBCA patterns were influenced by occasional transition-structure reminders (*SI Appendix*, *Supplementary Methods* and Fig. S11).

#### Converging evidence based on BA of parameters.

As explained above, to address participant heterogeneity we also ran our mixed-effects models (for MBCA and MFCA) for parameters that were estimated based on BA. For each participant, parameters were averaged across models with weights corresponding to posterior model probabilities (*SI Appendix*, *Supplementary Methods*). We found strong positive correlations (all *r* > 0.8) between the full model’s ML CA parameters and the corresponding BA parameters. Importantly, the mixed-effect models for the BA parameters supported the very same conclusions about CA in both systems (*SI Appendix*, Fig. S12).

##### The relationship between CA properties.

We explored the relationships between five measures of CA. For each participant, we calculated four measures based on the ML full-model parameters: overall levels of MBCA (an average of all four MBCA parameters), overall level of MFCA (an average of all four MFCA parameters), CM guidance to MFCA (the contrast between average MFCA parameters for related and unrelated outcomes), and importance-based modulation of MBCA (the contrast between average MBCA parameters for important and unimportant outcomes). The fifth measure was our model-agnostic measure of choice sensitivity. Pairwise correlation between these measures (*SI Appendix*, Fig. S13) revealed a positive correlation between MBCA and choice sensitivity (*r* = 0.34, *P* = 0.027), which is expected because, as we show below, MBCA is an effective way to learn to make good choices. Additionally, we found a positive correlation between the overall level of MFCA and CM guidance to MFCA (*r* = 0.38, *P* = 0.012). Neither of these correlations survived a Bonferroni correction for multiple (10 in this case) hypotheses.

### The Normative Status of CM Guidance to CA.

Our findings raise important questions regarding the effects of the reported modulations of CA (based on a CM) on the acquisition of reward. We therefore simulated agents performing our task under different regimes and over broad ranges of CA parameters (broader than the empirical ranges for MFCA and MBCA parameters in our task: −1.25 to 2.32 and −0.9 to 1.7, respectively; *SI Appendix*, *Supplementary Methods* for simulated parameter ranges). For each agent, we calculated a standardized measure of reward earnings defined as the excess earnings over a random guessing policy, relative to the excess earning of an omniscient “oracle agent” that is rewarded according to the expected reward of the better of the two bandits (*SI Appendix*, *Supplementary Methods*). We address MFCA and MBCA agents in turn (*SI Appendix*, *Supplementary Methods* for full simulation details).

#### CM guidance to MFCA enhances earnings.

**Fig. 5.**
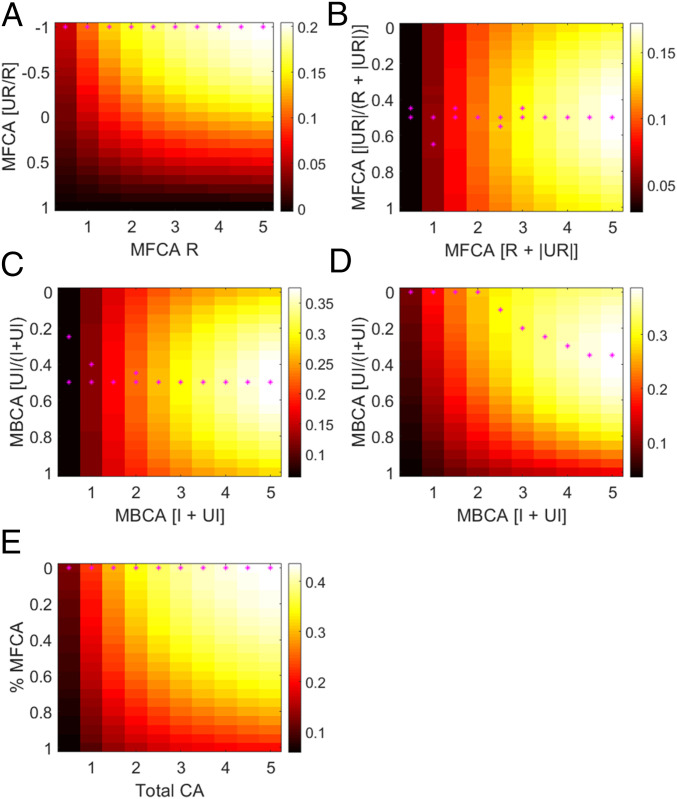
Simulated reward earnings for various regimes of CA parameters. (*A*) Standardized earning of simulated pure-MF agents (colorbar; *SI Appendix*, *Supplementary Methods*) are displayed as a function of MFCA for related (R) outcomes (abscissa; values greater than 0) and the ratio between MFCA for unrelated (UR) and related outcomes (ordinate). Earnings are maximized when the ratio is −1 (i.e., when MFCA for unrelated outcome is negative and equal in magnitude to the MFCA for related outcomes). (*B*) Standardized earning for pure-MF agents as a function of total MFCA for related and unrelated outcomes (abscissa) and the proportion of unrelated MFCA (ordinate). Here, MFCA is positive and negative for related and unrelated MFCA, respectively (hence an absolute value is applied to MFCA UR). Earnings are maximized when the proportion of MFCA for unrelated outcome is 0.5 (i.e., when related and unrelated MFCA are equal in magnitude and opposite in sign). (*C*) Standardized earning for pure-MB agents as a function of total MBCA for important (I) and unimportant (UI) outcomes (abscissa) and the proportion of unimportant MBCA (UI). Earnings are maximized when the proportion of MBCA for unimportant outcomes is 0.5 (i.e., when MBCA is equal for both outcome types). (*D*) Same as *C* but for a modified variant of the task wherein outcome importance is positively correlated across trials. (*E*) Standardized earning for hybrid MB–MF agents as a function of total CA (MB+MF; abscissa) and the proportion of MFCA (ordinate, which is equivalent to the proportion of MF control). Earnings are maximized when the proportion of MBCA is 1 (i.e., for pure reliance on MB control). Each data point in each panel is based on 10,000 simulations of synthetic experimental sessions. Magenta asterisks mark the maximal earning in each column. However, if the maximal earnings were not significantly higher than earnings in the central row of that column, we also marked the central row (hence some columns feature two asterisks).

To assess whether guidance from a CM is instrumental for MFCA, we simulated reward earnings for various regimes of MFCA. Fig. 5*A* displays standardized earnings of pure MFCA agents for different combinations of MFCA parameters for related (R: common = exclusive; *x* axis) and for the ratio between unrelated (UR: counterfactual = absent) and related outcomes (*y* axis). First, as expected, in the absence of guidance from a CM (bottom row of panel), when CA for related and unrelated outcomes are equal, earnings match those of a random choice policy (standardized earnings are 0) because the MF system lacks reliable information to distinguish between the values of the different individuals. As the unrelated MFCA parameters decrease (moving upward in the panel) manifesting guidance from a CM, standardized earnings increase. Earnings are maximized when these parameters are negative and equal in magnitude to the related MFCA parameters. Fig. 5*B* displays standardized earnings for a regime in which MFCA is positive and negative for related and unrelated outcomes, respectively. For any value of “total MFCA” (R+|UR|), earnings are maximized when all MFCA parameters are equal in magnitude. Thus, to the extent one relies on MFCA, guidance from a CM and negative CA from unrelated outcomes enhance earnings. We also found that MBCA is optimized when CA for related and unrelated outcomes are equal (*SI Appendix*, Fig. S14 *A* and *B*) (i.e., MBCA doesn’t benefit from modulation based on choice relatedness).

#### Importance-based MBCA is costly in our task.

Next, we asked whether importance-based modulation fosters earnings. Following the same design as Fig. 5*B*, Fig. 5*C* displays standardized earnings for pure MB agents for different combinations of MBCA parameters for important (I: exclusive = counterfactual) and unimportant (UI: common = absent) outcomes. For any value of “total MBCA” (I + UI), we find that earnings are maximized when the two MBCA parameters are equal (i.e., when the trade-off between important and unimportant MBCA is resolved in an equal split). Thus, importance-based modulation of MBCA is costly.

However, in each trial of our task, we present a random pair of individuals. Consequentially, current-state importance does not predict future-state importance. In more ecological environments, we might expect the importance of task aspects for decisions would enjoy positive temporal autocorrelation, such that currently important aspects are more likely also to be important for subsequent decisions. These are the very circumstances that importance-based modulation of MBCA should improve performance. Fig. 5*D* presents standardized earnings in a modified version of our task in which trial sequences were constructed such that outcome importance was positively autocorrelated across trials. As expected, we find standardized earnings are maximized when MBCA for important outcomes is higher than for unimportant outcomes. Thus, importance-based MBCA might reflect prolonged adaptation to more ecological environments. Finally, we also found that importance-based modulation of MFCA does benefit earning in our task (*SI Appendix*, Fig. S14 *C* and *D*). However, in the modified version of the task, an MFCA modulation based jointly on relatedness and importance boosts earnings (*SI Appendix*, Fig. S14 *E* and *F*).

#### MBCA outperforms MFCA in earnings.

Finally, we assess which of the two forms of CA is more instrumental for reward acquisition. Fig. 5*E* displays standardized earnings for hybrid agents who engage in both MB and MF control as a function of the “total CA” across both systems and the proportion of MFCA (which is equivalent to the relative reliance on MF control). For any value of total CA, earnings are maximized when the MF system is silent, that is, when the trade-off between the systems is resolved in pure reliance on MB control.

In summary, we find that MB control outperforms MF control with respect to earnings. However, to the extent that one relies on MF control and guidance from a CM, manifesting in negative CA for unrelated outcomes is beneficial. Furthermore, whereas importance-based modulation of MBCA is inefficient in our task, it can be instrumental in environments characterized by the temporal consistency of important task states. We discuss these findings in greater detail below.

## Discussion

We recently described a dual-outcome bandit task (25) that distinguishes holistic information about a choice (identified here with the person) from more granular information pertaining to outcomes of that choice (identified with the vegetables). This task allows us to dissociate MB from MF influences on decisions, enabling an examination of the influence of knowledge inherent in a CM (30–33), over MBCA and MFCA. Here, using a version of the task in which full feedback about outcomes is provided, we examined whether, and how, CM-based processes resolve this overexuberant information by modulating MF and MB learning.

We first show that a CM guides MFCA. This supports the notion that an MF system, which retrieves cached action values during choice without reference to task transition structure can, when assigning credit to past actions, be guided by a CM. Many previous experimental investigations simplified greatly the problem of associating actions with outcomes, for instance, by providing relevant rewards and/or state information immediately after each action. In such cases, MFCA can exploit factors such as temporal contiguity (26), rendering CM-based guidance nugatory. CM-based guidance for MFCA, however, becomes crucial in complex real-life situations when reward feedback pertaining to a past action is either delayed or promiscuously available. Without such guidance, an attribution of outcomes is impossible.

In the context of our task, in the absence of CM guidance, MFCA must be equally affected by choice-related and -unrelated outcomes. In striking contrast to this, we found that action-related and -unrelated outcomes reinforced MF action values positively and negatively, respectively. In our computational treatment, CM-based guidance is achieved by modulating the extent of updates of MF values based on outcomes according to the outcome structure of actual choices. One could equally imagine the operation of an architecture such as DYNA (9, 34), in which the MB system trains an MF system by generating offline, hypothetical, replay-, or preplay-based (35–37) MB episodes from which an MF system learns as if they were actualized. Notably, had the MF system been fully trained by DYNA, then it would have mimicked perfectly the MB system and we would not have found evidence for MFCA effects.

The negative choice-unrelated MFCA effect might reflect an MF attempt to learn relative rather than absolute action values (38). Conceivably, action-unrelated rewards serve to signal that one’s chosen action may be inferior to other potential actions. Hence, an MF penalty is imposed on the current action—a process that can boost exploration of alternative actions. This could operate in the absence of MB inference as to which actions led to these unrelated rewarding outcomes. Alternatively, negative choice-unrelated MFCA might reflect a process by which choice-unrelated rewards contextualize choice-related rewards, akin to divisive normalization (39). According to this account, MFCA is driven solely by choice-related outcomes, but those related outcomes are conceived as more/less valuable when unrelated outcomes are unrewarded/rewarded. Future research should examine the cognitive underpinnings and the neural implementation of this negative reinforcement.

By stark contrast with updates to MF values, MB values were affected almost equally by outcomes that were related, or unrelated, to choice. However, unlike MF values, MB updates were seemingly affected by the decision-making processes associated with a choice, with a greater effect seen for outcomes a CM could discriminate as being determinative (Exclusive and Counterfactual) compared to those that were not (Common and Absent). We suggest this fits with the idea that MB deliberation differentially activates, or primes, components of a CM based on their “importance” and that such state activation facilitates CA. Hypothetically, other variables, such as the number of times a state was incorporated into previous action plans, could similarly contribute to a gradient of activation over a CM. Future studies should examine which variables affect state activation and how they affect the extent of CA. A rather different possibility is that “importance”-based MBCA is mediated by an attentional process whereby important outcomes capture more attention during outcome presentation. Again, it will be of interest to probe these attentional influences on MBCA, for example, by presenting outcomes in parallel rather than serially and measuring the level of attention allocation to different outcomes (40, 41).

Our findings raise subtle questions as to the benefits of the observed CM-based modulations of MBCA and MFCA. Using model simulations, we find a rich picture. First, to the extent that one relies on MFCA, guidance from a CM that manifests in negative CA for choice-unrelated outcome is instrumental for simulated earnings. Furthermore, we found that simulated earnings were maximal when MFCA for related and unrelated outcomes were equal in magnitude but reversed in sign. Empirically, however, the positivity of MFCA to related outcomes in our task was larger than the negativity of MFCA to unrelated outcome (beta = 0.23, *t*(164) = 6.28, *P* = 3 × 10^−9^; Fig. 4*B*), suggesting participants relied too little on negative MFCA to unrelated outcomes.

However, we also found that, as is the case in some variants of the two-step task (42, 43), reliance on MB control in our task is more profitable than MF control. Nevertheless, a conclusion that reliance on the latter is nonnormative is premature given the relative cognitive costs and/or computational accuracy of MF control (44). Furthermore, although importance-based modulation of MBCA is not instrumental to profits in our task, it is helpful in more ecological environments where the importance of task states is temporally consistent (rather than being random as in our task). In such instances, state importance signals a need to maintain more accurate state-value representations (45). Thus, to the extent that CA for important and unimportant outcomes trade off, it should be diminished for “unimportant” relative to “important” outcomes.

We acknowledge that the various CM-based influences on MFCA and MBCA found at a group level will not necessarily all co-occur within individual participants. Indeed, our Bayesian analysis of model prevalence revealed that about 70% of the population is expected to engage in either pure-MFCA or pure-MBCA processing. Future studies can help illuminate how agents arbitrate between the various CM-based strategies as well as the situational variables (e.g., time pressure during choice and/or during feedback) which might influence this arbitration and the expression of individual difference in strategy selection.

In sum, our results encourage a generalized view of model-based processing. That is, instead of focusing narrowly on the sort of prospective reasoning of the sort that underlies planning, it suggests a broader perspective on the types of operations supported by a CM, where these include retrospective inference of state uncertainty (25) and the filtering of online experiences. Our findings suggest that a CM which exploits the full range of these operations can shape best the precise impact of reward feedback. A critical next step is to characterize and quantify the cognitive costs incurred by MB and MF control as well as by CM-based modulation of learning. These will allow a better understanding of the cognitive process underlying arbitration between these control strategies and will facilitate a development of a framework to assess the benefits and beneficial interaction of these processes.

## Methods

### Participants.

A total of 44 participants were recruited from the SONA subject pool (https://uclpsychology.sona-systems.com/Default.aspx?ReturnUrl=/) with restrictions of having normal or corrected vision, being nondyslexic, being a UK-based student, and being born after 1981. The study was approved by the University College London Research Ethics Committee (Project ID 9929/002). Subjects gave written informed consent before the experiment.

### Experimental Procedures.

Participants were first familiarized with four pictures of persons and learned which pair of vegetables each person grows [pictures of persons and vegetables were adopted from previous studies (46, 47)]. Each vegetable was grown by two different individuals, and each person grew a unique pair of vegetables. The mapping between persons and vegetables was created randomly anew for each participant and remained stationary throughout the task. After learning, participants were quizzed about which vegetables each person grew and about which person they would choose to obtain a target vegetable. Participants iterated between learning and quiz phases until they achieved perfect quiz performance (100% accuracy and RT [reaction time]) < 3,000 ms for each question).

After learning, participants played eight practice-bandit trials to verify that they understood the task. These practice trials proceeded as described below with the sole difference that no time limit was imposed on a choice. They next played seven blocks, each comprising 56 bandit trials. On each trial, a pair from the four persons were offered for choice, and participants had 750 ms to choose one of these objects (left or right mouse click). Offered persons always shared one vegetable in common. This defined four person pairs, each presented on 14 trials (per block) in a random order. Following a choice, participants saw sequentially, and in random order, whether each of the four vegetables that the chosen person grows was sold or not. Participants earned a notational £1 per sale of each of the two vegetables that were grown by the person they chose (they earned no money for the two nonrewarded vegetables). Importantly, the feedback stage indicated to participants neither which pair of vegetables were grown by their chosen person nor their earning on that trial. The sale probabilities (“demands”) of the four vegetables evolved across trials according to four independent Gaussian-increment random walks with reflecting boundaries at *P* = 0.2 and *P* = 0.8 and an SD of 0.03 per trial. A new random walk was created for each block of trials, and the four random walks were initialized with a random permutation of (0.2, 0.4, 0.6, and 0.8).

After each block was completed, participants had a forced 1-min (minimum) break. After the break, participants were informed that all vegetable reward probabilities were reset to new values, and, therefore, they should form new impressions of these when the task resumes. Additionally, at the beginning of blocks 1 and 4, participants received refresher training on the transition structure.

The task lasted about 60 min. Participants were paid £8 per hour plus a performance-based bonus, which was calculated based on the total amount of earned rewards on three randomly sampled trials.

### Data Analysis.

Two participants were excluded from data analyses due to disengagement with the task. One participant used the same response key for 98% of the trials, and the other switched the response key on 86% of trials. The remaining 42 participants were the targets for the analysis.

### Model-Agnostic Analysis for MB and MF Contribution to Choice.

Our model-agnostic analyses were conducted using logistic mixed-effect models (implemented with MATLAB’s function “fitglme”) with participants serving as random effects with a free covariance matrix. In our first analysis, we used only trials n+1 that offered for choice the trial n–chosen person. Our regressors COMMON (Common vegetable), UNIQUE (Unique vegetable), ABSENT (Absent vegetable), and OTHER (Other vegetable) coded whether trial n outcomes were rewarding (coded as +0.5 for reward and −0.5 for nonreward); the regressor IMPORTANCE coded whether the UNIQUE and OTHER outcomes were important on the previous trial n (+0.5 for important and −0.5 for unimportant); and the regressed variable REPEAT indicated whether the choice on the focal trial n+1 was repeated. PART coded the participant contributing each trial. The model, in Wilkinson notation, was REPEAT ∼ IMPORTANCE × (COMMON + UNIQUE + ABSENT + OTHER) + (IMPORTANCE × (COMMON + UNIQUE + ABSENT + OTHER)|PART). We used an F-test to examine contrasts on fixed effects.

We performed an additional analysis relying on all trial-to-trial transitions. On each trial (n+1), the two offered persons shared one vegetable in common, and each had one unique vegetable. The regress variable CHOOSE_RIGHT indicated whether the person offered on the right side of the display was chosen (trial n+1), and our regressors were R1 and R2, coding whether the unique vegetables of the right-side and left-side persons were rewarded on the previous trial n (coded as +/−0.5), and IMPORTANCE, coding whether these two vegetables were important on trial n (coded as ±0.5). PART coded the participant contributing each trial. The model, in Wilkinson notation, was CHOOSE_RIGHT ∼ IMPORTANCE × (R1 + R2) + (IMPORTANCE × (R1 + R2)|PART). We used an F-test to examine whether any of the fixed-effects interactions with importance were significant. If this F-test was significant, we proceeded to examine the significance of the individual interaction terms (R1 × IMPORTANCE, R2 × IMPORTANCE). Similarly, we used F-tests to examine whether any of the reward effects were significant. If the F-test was significant, we examined the significance of the individual terms (R1, R2).

### Computational Models.

We formulated a hybrid RL model to account for the series of choices for each participant. In the model, choices are contributed by both the MB and MF systems. The MF system caches a $Q^{\text{MF}}$ value for each person, subsequently retrieved when the person is offered for choice. During learning, following reward feedback, rewards from the various vegetables (Common, Exclusive, Counterfactual, and Absent; these assignments are the contribution of MB information to MFCA) are used to update the $Q^{\text{MF}}$ value for the chosen person as follows:

$$Q^{\text{MF}}\left(\text{chosen}\;\text{person}\right)\leftarrow \;\left(1-f^{MF}\right)*Q^{\text{MF}}\left(\text{chosen}\;\text{person}\right)+c_{common}^{MF}*r_{common}+c_{exclusive}^{MF}*r_{exclusive}+\;c_{counter}^{MF}*r_{counter}+c_{absent}^{MF}*r_{absent},$$ [2]

where the “c”s are four free MFCA parameters corresponding to the four outcome types, the “r”s are the rewards for the four outcome types (codes as 1 for reward or −1 for nonreward), and $f^{MF}$(between 0 and 1) is a free parameter corresponding to forgetting in the MF system. The MF values of the three nonchosen persons were subject to forgetting:

$$Q^{\text{MF}}\left(\text{non}\;\text{choen}\;\text{person}\right)\leftarrow \left(1-f^{MF}\right)*Q^{\text{MF}}\left(\text{non}\;\text{chosen}\;\text{person}\right).$$ [3]

Unlike MF, the MB system maintains $Q^{\text{MB}}$ values for the four different vegetables. During choices, the $Q^{\text{MB}}$ value for each offered person is calculated based on the transition structure:

$$Q^{\text{MB}}\left(\text{person}\right)=Q^{\text{MB}}\left(\text{veg}\;1\right)+Q^{\text{MB}}\left(\text{veg}\;2\right).$$ [4]

Following a choice, the MB system updates the $Q^{\text{MB}}$ values of each of the four observed vegetable based on its own reward:

$$Q^{\text{MB}}\left(\text{vegetable}\right)\leftarrow \;\left(1-f^{MB}\right)*Q^{\text{MB}}\left(\text{vegetable}\right)+c_{type}^{MB}*r_{type},$$ [5]

where $f^{MB}$ (between 0 and 1) is a free parameter corresponding to forgetting in the MB system, “type” is the vegetable’s outcome type designation (Common, Exclusive, Absent, or Counterfactual), and $c_{common}^{MB},c_{exclusive}^{MB},c_{counter}^{MB},c_{absent}^{MB}$ are four free MBCA parameters.

Our model additionally included progressive perseveration for chosen persons. After each trial, the perseveration values of each of the four persons updated according to

$$PERS\left(\text{person}\right)\leftarrow \left(1-f^{P}\right)*PERS\left(\text{person}\right)+\text{pr}*1_{\text{person}=\text{chosen}},$$ [6]

where $1_{\text{person}=\text{chosen}}$ is the chosen person indicator, pr is a free perseveration parameter, and $f^{P}$(between 0 and 1) is a free perseveration-forgetting parameter.

During choice, a net Q value was calculated for each offered person:

$$Q_{\text{net}}\left(\text{person}\right)=Q^{\text{MB}}\left(\text{person}\right)+Q^{\text{MF}}\left(\text{person}\right)+PERS\left(person\right).$$ [7]

The $Q_{\text{net}}$ values for the two persons offered for choice are then injected into a softmax choice rule such that the probability to choose an option is as follows:

$$Prob\left(\text{person}\right)=\frac{e^{Q_{\text{net}}\left(\text{person}\right)}}{e^{\left[Q_{\text{net}}\left(\text{person}\right)+Q_{\text{net}}\left(\text{other}\;\text{person}\right)\right]}}.$$ [8]

$Q^{\text{MF}}$ and PERS person values and $Q^{\text{MB}}$ vegetable values were initialized to 0 at the beginning of each experimental block.

## Supplementary Material

Supplementary File

## Data Availability

The data that support the findings of this study and data analysis code have been deposited in the Open Science Framework (OSF) and are available in the following link: https://osf.io/ydrv7/?view_only=c0c00083077d45e18f7997d53172150e.
