# Supplementary material for: Human subjects exploit a cognitive map for credit assignment
Source: Proc Natl Acad Sci U S A. 2021 Jan 21;118(4):e2016884118. doi: 10.1073/pnas.2016884118 (PMC7848688; doi:10.1073/pnas.2016884118)
Supplement: Supplementary File [file pnas.2016884118.sapp.pdf]

PNAS
www.pnas.org

Supplementary Information for
Human subjects exploit a cognitive map for credit assignment.

Rani Moran, Peter Dayan*, Raymond J. Dolan*

Rani Moran
Email: r.moran@ucl.ac.uk

This PDF file includes:

Supplementary text
Figures S1 to S15
Tables S1
SI References

Supplementary Information Text

Supplementary Methods

Model Fitting and Model Comparison

We fit our choice models to the data of each individual, maximizing the likelihood (ML) of their choices (we optimized likelihood using MATLAB's 'fmincon', with 200 random starting points per participant; Table S1 for best-fitting parameters). Our full hybrid agents, which allowed for contributions from both an MB and an MF system, served as a super-model in a family of five nested sub-models: 1) a pure MBCA agent, eliminated MFCA by constraining all MF parameters to 0: $c^{MF}_{common} = c^{MF}_{exclusive} = c^{MF}_{absent} = c^{MF}_{counter} = f^{MF} = 0$, 2) 1) a pure MFCA agent, eliminated MBCA by constraining all MB parameters to 0: $c^{MB}_{common} = c^{MB}_{exclusive} = c^{MB}_{absent} = c^{MB}_{counter} = f^{MB} = 0$. Note that while MB didn't assign credit to vegetables it was still allowed to guide MF credit assignment to persons, 3) a no CM guidance for MBCA agent that constrained equality between the four MFCA parameters, $c^{MF}_{common} = c^{MF}_{exclusive} = c^{MF}_{absent} = c^{MF}_{counter}$, 4) an egalitarian MBCA agent, which constrained equality between the 4 MBCA parameters $c^{MB}_{common} = c^{MB}_{exclusive} = c^{MB}_{absent} = c^{MB}_{counter}$ and 5) a no MFCA to choice unrelated outcomes, which constrained the MFCA to choice-unrelated outcomes to 0, $c^{MF}_{absent} = c^{MF}_{counter} = 0$.

We next conducted a bootstrapped generalized likelihood ratio test (BGLRT[1]) for the super-model vs. each of the sub-models separately. In a nutshell, this method is based on the classical-statistics hypothesis testing approach and specifically on the generalized-likelihood ratio test (GLRT). However, whereas GLRT assumes asymptotic Chi-squared

null distribution for the log-likelihood improvement of a super model over a sub-model, in BGLRT these distributions are derived empirically based on a parametric bootstrap method. In each of our model comparison the sub model serves as the H0 null hypothesis whereas the full model as the alternative H1 hypothesis.

For each participant, we created 1001 synthetic experimental sessions by simulating the sub-agent with the ML parameters on novel trial sequences which were generated as in the actual data. We next fitted both the super-agent and the sub-agent to each synthetic dataset and calculated the improvement in twice the logarithm of the likelihood for the full model. For each participant, these 1001 likelihood-improvement values served as a null distribution to reject the sub-model. The p-value for each participant was calculated based on the proportion of synthetic dataset for which the twice logarithm of the likelihood-improvement was at least as large as the empirical improvement. Additionally, we performed the model comparison at the group level. We repeated the following 10,000 times. For each participant we chose randomly, and uniformly, one of his/her 1,000 synthetic twice log-likelihood super-model improvements and we summed across participant. These 10,000 obtained values constitute the distribution of group super-model likelihood improvement under the null hypothesis that all participants rely on a sub-model. We then calculated the p-value for rejecting the sub-agent at the group level as the proportion of synthetic datasets for which the super-agent twice logarithm of the likelihood improvement was larger or equal to the empirical improvement in super-model, summed across participants. Note rejecting the sub-model at the group level supports an inference that a subset of participants rely on the sub-model

but it does not imply the full model is more prevalent in the subject-population (We used a different approach for assessing the prevalence of the various sub-models- see below).

We also used an iterated batch importance sampler (IBIS) algorithm[2] to perform Bayesian model-fitting (using implementation methods as suggested by Findling and colleagues[3]) for each participant. We used uniform priors for all parameters with range [-2 2] for CA parameters, [0-1] for forgetting (CA and perseveration), and [-1.5 1.5] for the perseveration parameter. Using IBIS, we computed the posterior mean parameters and marginal likelihood (model evidence) for each participant x model.

Bayesian Averaging of Model Parameters

For each participant we calculated Bayesian Averaged parameters as follows. Assuming a uniform prior over models in our model 6-model family, the posterior probability of each model is proportional to its model evidence (obtained using the IBIS algorithm). Thus, the posterior probability of a model is its evidence divided by the sum model-evidence across all 6 models. These posterior probabilities served as weights for averaging parameters across models. In order to average parameters across models, parameter vectors from sub-models were augmented to constitute parameter vectors of the full model (for example, parameter vectors from the pure MBCAMFCA sub-models were augmented with four MBCA/MBCA parameters equal to 0).

Assessing model Prevalence and Exceedance Probabilities.

To assess model-prevalence we used a hierarchical Bayesian approach for model selection[4,5]. This method treats models as random effects which may vary across

participants. It provides a posterior Dirichlet distribution over models. Each model's normalized posterior concentration parameter (i.e., its concentration parameters divided by summed concentrations across models) constitutes the expected model probability in the population (Fig. 4F). This method also prescribes the calculation of protected exceedance probabilities (i.e., the probability that each model is the most prevalent in the model-set taking into account that apparent differences in model prevalence may be due to chance while in truth all models are equally prevalent).

Model Recovery

Because our model-comparisons are based on BGLRT, model recovery questions are tantamount to assessing type-I error rates and power. We used a type I error rate of .05 meaning that by design, if a null model (one of our sub-models) generated the data it will not be rejected (i.e., it will be "recovered") with a probability of .95. To estimate the power of our design, we repeated the same steps as in our BGLRT analysis, but this time using synthetic data that was simulated from the full model instead of the sub-models. For each simulated data set, we examined whether each of the null models were rejected at the group-level, according to BGLRT, in favour of the full model. We found that for all 10,000 simulations, all sub models were rejected in favour of the full model (i.e., the full model was "recovered") at the group level. Thus the estimated power is very close to 1 for assessing all sub-models.

Model Simulations

To generate model predictions (Figs. S2-S3) with respect to choices, we simulated for each participant, 25 synthetic experimental sessions (novel trial sequences were generated as in the actual experiment), based on his or her ML parameters obtained from

the corresponding model fits (the models are described above). We then analysed these data in the same way as the original empirical data (but with datasets that were 25 times larger, as compared to the empirical data, per participant). Once more, while this method of replicating participants allows us to reduce noise from a calculation of model predictions, it does not allow us to examine how likely model-agnostic signatures of interest emerge in model-simulations for synthetic data of the same size as the empirical dataset. Thus, for Fig. S4 we followed the same procedure but with a single synthetic experimental session per participant. This procedure was repeated 1,001 and we calculated the proportion of simulations in which fixed effects of interest were significant.

Analysis of Model parameters

For each participants we obtained, based on the full model, four MBCA and four MFCA maximal-likelihood (ML) parameter estimates corresponding to the four outcome types (common, exclusive, counterfactual, absent). For each system separately we ran a mixed effects model (implemented with MATLAB's function "fitglme") for the CA parameters (denoted C), with participants (PART) serving as random effects with a free covariance matrix. Our regressor where Importance (denoted I; coded as +.5 for the exclusive and counterfactual outcomes and as -.5 for the common and absent outcomes), and choice-Relatedness (denoted R; coded as +.5 for the exclusive and common outcomes and as -.5 for the counterfactual and absent outcomes). The model, in Wilkinson notation, was: C~ I*R + (I*R|PART). We also ran our mixed effects models for BA, rather than ML parameters (see above).

For the analysis reported in Fig S11, we used ML parameters and included a transition structure reminder (T) indicator (corded as +.5 for experimental blocks 1 and 4, which included transition structure-reminders and as -.5 for the remaining blocks): C~ I*R*T + (1|PART).

Parameter Recovery and Trade-offs

We tested parameter recovery of our full model based on the following method. For each participant, we create 1,000 synthetic datasets by simulating the full model based on his/her best fitting ML parameters. We then fitted these datasets with the full model (once more using ML-estimation). We assessed the Pearson correlations between each generating parameter and the corresponding recovered parameter across these 42*1000 datasets (Fig. S5). We duplicated participants to remove noise from the calculation of the correlations expected at the population level.

Additionally, for each parameter and each synthetic dataset (of 42,000 datasets) we calculated the estimation error as the difference between the generating parameter and the recovered parameter. We assessed parameter trade-offs by calculating Pearson correlations between estimation errors for each pair of parameters (Fig. S7).

Similarly, based on our parameter recovery simulation, we also assessed the full model's recovery of the relatedness effect on MFCA ($.5 * (c^{MF}_{common} + c^{MF}_{exclusive} - c^{MF}_{absent} - c^{MF}_{counter})$) and the importance effect on MBCA ($.5 * (-c^{MB}_{common} + c^{MB}_{exclusive} - c^{MB}_{absent} + c^{MB}_{counter})$). We calculated Pearson correlations between generating and estimated effects. Finally, the trade-off between these two effects was calculated as the Pearson correlation between the estimation errors of these two effects (Fig. S9A-B). For

Figure S10B we calculated the correlation between estimation-errors for $(c^{MB}_{exclusive} - c^{MB}_{common})$ and $(c^{MF}_{counter} - c^{MF}_{absent})$.

While the above analyses are informative for assessing the properties of parameter-estimation for individual parameters, our primary current interest is on assessing group level-effects. Thus, we also tested parameter-recovery at the level of the group-averages, rather than individual participants. We first bootstrapped 42 out of the (42 x 1000) synthetic datasets described above and we repeated the same analyses but focusing on the averaged parameters across these 42 sampled datasets. This entire procedure was repeated 1,000 times (Figs. S6, S8, S9C-D).

Simulations of reward-earnings

We simulated 10,000 agents for each of several combinations of MFCA and MBCA parameters on synthetic experimental sessions that were constructed in the same way as the empirical sessions (except for the case of Fig. 5D, see below). For each agent, we calculated, the average number of points it earned per-trial based on its simulated choices and consequent rewards. We compared these average earnings to those on the same experimental trials of two, yoked, additional agents: ‘guessing’ and ‘oracle’. On each trial, the guessing agent earned the average expected total reward (according to the generating outcome-reward probabilities) provided by both bandits, whereas the oracle agent omnisciently earned the maximal total expected reward across the two offered bandits. For comparison, we calculated the standardized earnings of the MBCA agent as the ratio of how much more it earned than the guessing agent to how much more the oracle agent earned than the guessing agent. In all simulations the forgetting rates were set to .5, which is similar to the average group values ($f^{MB} = f^{MF} = .5$) and agents

expressed no choice-perseveration tendencies ($\text{pr} = f^P = 0$). The parameter combinations we used is the various simulations displayed in Fig. 5 are detailed below.

For Fig. 5A-B, we simulated pure MFCA agents. For Fig. 5A, the MFCA parameter for related outcomes ($c_{related}^{MF} \triangleq c_{common}^{MF} = c_{exclusive}^{MF}$) varied between 0.5 and 5 in steps of 0.5. The MFCA parameter ($c_{unrelated}^{MF} \triangleq c_{counter}^{MF} = c_{absent}^{MF}$) varied as ($c_{unrelated}^{MF} = \alpha c_{related}^{MF}$) where α varied between -1 to 1 in steps of 0.1. For Fig. 5B, the total MFCA for related and unrelated outcomes ($c_{total}^{MF} \triangleq c_{related}^{MF} - c_{unrelated}^{MF}$) varied between 0.5 and 5 in steps of 0.5. For each value of total MFCA we examined combinations where $c_{unrelated}^{MF} = -\alpha c_{total}^{MF}$ and $c_{related}^{MF} = (1-\alpha) c_{total}^{MF}$ where α varied between 0 and 1 in steps of 0.05. Note that for panel B, MFCA parameters for related and unrelated outcomes were always non-negative and non-positive, respectively.

For Fig. 5C, we simulated pure MBCA agents as follows: the total MBCA for important ($c_{important}^{MB} \triangleq c_{exclusive}^{MB} = c_{counter}^{MB}$) and unimportant outcomes ($c_{unimportant}^{MB} \triangleq c_{common}^{MB} = c_{absent}^{MB}$) varied between 0.5 and 5 in steps of 0.5 ($c_{total}^{MB} \triangleq c_{important}^{MB} + c_{unimportant}^{MB}$). For each value of total MBCA we examined combinations where $c_{important}^{MB} = \alpha c_{total}^{MB}$ and $c_{unimportant}^{MB} = (1-\alpha) c_{total}^{MB}$ where α varied between 0 and 1 in steps of 0.05.

Fig. 5D was similar to Fig. 5C, but with a single important difference. Unlike the empirical experimental sessions, here we constructed trial sequences in which importance was auto-correlated. Specifically, there was a .8 (or .2) probability that the two important outcomes on one trial were also important (or unimportant) in the immediately preceding trial.

For Fig. 5E, we simulated hybrid agents that relied on both MBCA and MFCA. Each system was configured to its most profitable variant, that is, MBCA was equal for important and unimportant outcomes ($c^{MB} \triangleq c^{MB}_{important} = c^{MB}_{unimportant}$) and MFCA was equal in magnitude and opposite in sign for related and unrelated outcomes ($c^{MF} \triangleq c^{MF}_{related} = -c^{MF}_{unrelated} \geq 0$). Total CA across both systems varied between 0.5 and 5 in steps of 0.5 ($c_{total} \triangleq c^{MB} + c^{MF}$). For each value of total CA we examined combinations where $c^{MB} = \alpha c_{total}$ and $c^{MF} = (1-\alpha)c_{total}$ where α varied between 0 and 1 in steps of 0.05.

For Fig. S14A-B we repeated the simulations in Fig. 5A-B but for MBCA instead of MFCA. For Fig. S14C-D we repeated the simulations in Fig. 5C-D but for MFCA instead of MFBA. Finally, the simulations for Fig. S14 E-F were similar to the corresponding simulations for Fig S14C-D but MFCA for unrelated outcomes were set to minus the corresponding related- MFCA parameters. To elaborate, the total MFCA for important ($c^{MF}_{important} \triangleq c^{MF}_{exclusive} = -c^{MF}_{counter} \geq 0$) and unimportant outcomes ($c^{MF}_{unimportant} \triangleq c^{MF}_{common} = -c^{MF}_{absent} \geq 0$) varied between 0.5 and 5 in steps of 0.5 ($c^{MF}_{total} \triangleq c^{MF}_{important} + c^{MF}_{unimportant}$). For each value of total MFCA we examined combinations where $c^{MF}_{important} = \alpha c^{MF}_{total}$ and $c^{MF}_{unimportant} = (1-\alpha)c^{MF}_{total}$ where α varied between 0 and 1 in steps of 0.05.

Calculating Choice Accuracy for an Optimal Bayesian Learner

We simulated an optimal Bayesian learner on the empirical experimental sessions and calculated its accuracy rate (i.e., proportion of choices of the person with a higher generating expected reward, defined as the mean of the generating reward probabilities

across the associated vegetable pair). At the beginning of each block, the learner was initialized with four uniform belief distributions U[0.2, 0.8] pertaining to the reward probabilities of the four vegetables.

During the choice stage (on trial t), the learner calculated the expected reward (ER) for each vegetable (v) as $ER(v) = \int_{0.2}^{0.8} b_t(p; v) p dp$, where $b_t(p; v)$ denotes the belief distribution for vegetable v. Next, the learner calculated the expected reward for each offered person as a sum of ER's over the vegetable pair associated with that person. The learner then choses the person with the higher expected reward.

During the feedback stage the belief distribution for each vegetable was updated in 3 steps: 1) The observer used Bayes rule to update the belief about vegetable b given reward feedback as: $b_{t+1}(p; v) \propto p^{r_v}(1-p)^{1-r_v} b_t(p; v|r_v)$, where r_v denotes the reward (0 or 1) for vegetable v on trial t. 2) the belief distribution was convoluted with a zero-centred Gaussian with std= .03 to account for the random-walk evolvement of reward probabilities: $b_{t+1}(p; v) \leftarrow conv(b_{t+1}(p; v), N(0, .03^2)$. 3) the resulting belief was "folded" and truncated to the range [0.2., .8], corresponding to reflecting random walk boundaries (e.g., the probability density for p=.13 was added to p=.27). The resulting belief distribution governed choice on the following trial t+1.

Modelling Choices

Here we clarify our modelling choices. First, it is important to note that our forgetting parameters can be rewritten in terms of a standard (Rescorla-Wagner) form

using learning rates. To see this consider a Q-value update of the form we use in our models (henceforth the “ca parametrisation”):

$$(Eq.\ S1) \quad Q \leftarrow (1-f) * Q + ca$$

This update can be equivalently written as (henceforth the “r parametrisation”):

$$(Eq.\ S2) \quad Q \leftarrow Q + f * \left(\frac{ca}{f} - Q\right) = Q + f * (r - Q)$$

Where r= ca/f. Eq. (S2) is of the Rescorla-Wagner form, in which f serves as a leaning rate.

Thus, instead of using the “ca parametrisation” we could equivalently have used the “r parametrisation” and estimated free “r” (rather than “ca”) parameters to quantify the sensitivity of each system (MB and MF) to rewards provided to the various outcome types (common/exclusive/counter-factual/absent). In other words, the difference between our ca-formulation and a more “standard” r-formulation is not in the absence of learning rates, but rather whether ca or r parameters are used.

Prior to addressing why we opted for the “ca” rather than the “r” parameterisation, it is important to note that neither of these formulations renders it possible to identify[6] these parameters (either ca of r) separately from “decision noise” i.e., soft-max temperature parameters. For example, had our model included a MB soft-max temperature parameter then doubling all the MB ca (or r) parameters and the choice temperature would have maintained model-predictions invariant. Thus, a scaling of the model is mandatory. Because our main focus here is on a composition of credit assignment effects, we used the soft-max temperature as a scaling parameter and fixed it to 1. Admittedly this is arbitrary (as any scaling would be) but it useful as it allows us to

identify credit-assignment parameters for the various outcomes as well as allows us to compare them.

In passing, we think it helpful and important, to point out that a similar scaling problem affects almost all applications of RL models. Consider for example a simple two arm bandit task wherein each bandit provides a (1£) reward with a Bernoulli probability. Often, in models of this task a modeller will simply define outcomes as r=1 (Reward) and r=0 (non-reward), a practice that allows estimating a decision noise parameter (and a learning rate). Critically, this formulation assumes the subjective value of the reward (or non-reward) is equal for all participants. This assumption is likely to be wrong (e.g., 1£ may subjectively worth more to poorer participants) but is commonly used as a form of scaling.

Finally, the reason we preferred the "ca" over the "r" parameter is that we find it in practice to be more stable. When we fit the model using the "r" notation we find in practice that it difficult to identify f and r separately. In many cases, there are substantial trade-offs between r and f such that f takes extremely small values (very close to 0) while simultaneously, r takes huge values, which are problematic for interpretation and for statistical inferences. However, their multiplication r*f is still identifiable. Note that in our task this multiplication simply corresponds to f*r= f*ca/f= ca. Thus "ca" yield more stable and interpretable parameter estimates (See Fig. S15 for illustration).

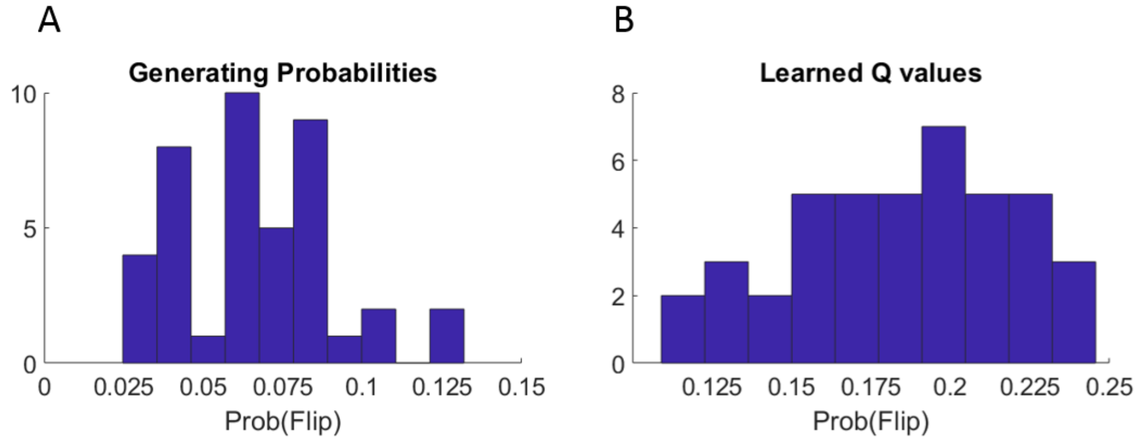

Fig S1. Related to Fig. 1. Changes in value-rankings of offered individuals. A) We calculated for each participant the proportion of trials in which the identity of the more rewarding of the two offered individuals (based on the average generating random-walk reward-probabilities of vegetable-pairs associated with each person), flipped relative to the previous occurrence of the same trial in the same experimental block (x-axes). The histogram of these flip-probabilities is shown. B) Participants do not have access to generating vegetable probabilities, but only to impoverished binary reward-feedback. Hence, they have to learn vegetable reward-probabilities. Here we simulated Q-value learning for vegetables (learning rate = .4, which is similar to the average learning rate we estimate) and calculated the same flipping probabilities but based on person- MB Q values, rather than generating probabilities (a person's value is the sum of the Q-values of the associated vegetable pair). For such learners ranking-flips will seem very frequent (for a more modest learning rate of .2, the average proportion of flip trials was ~ 11%).

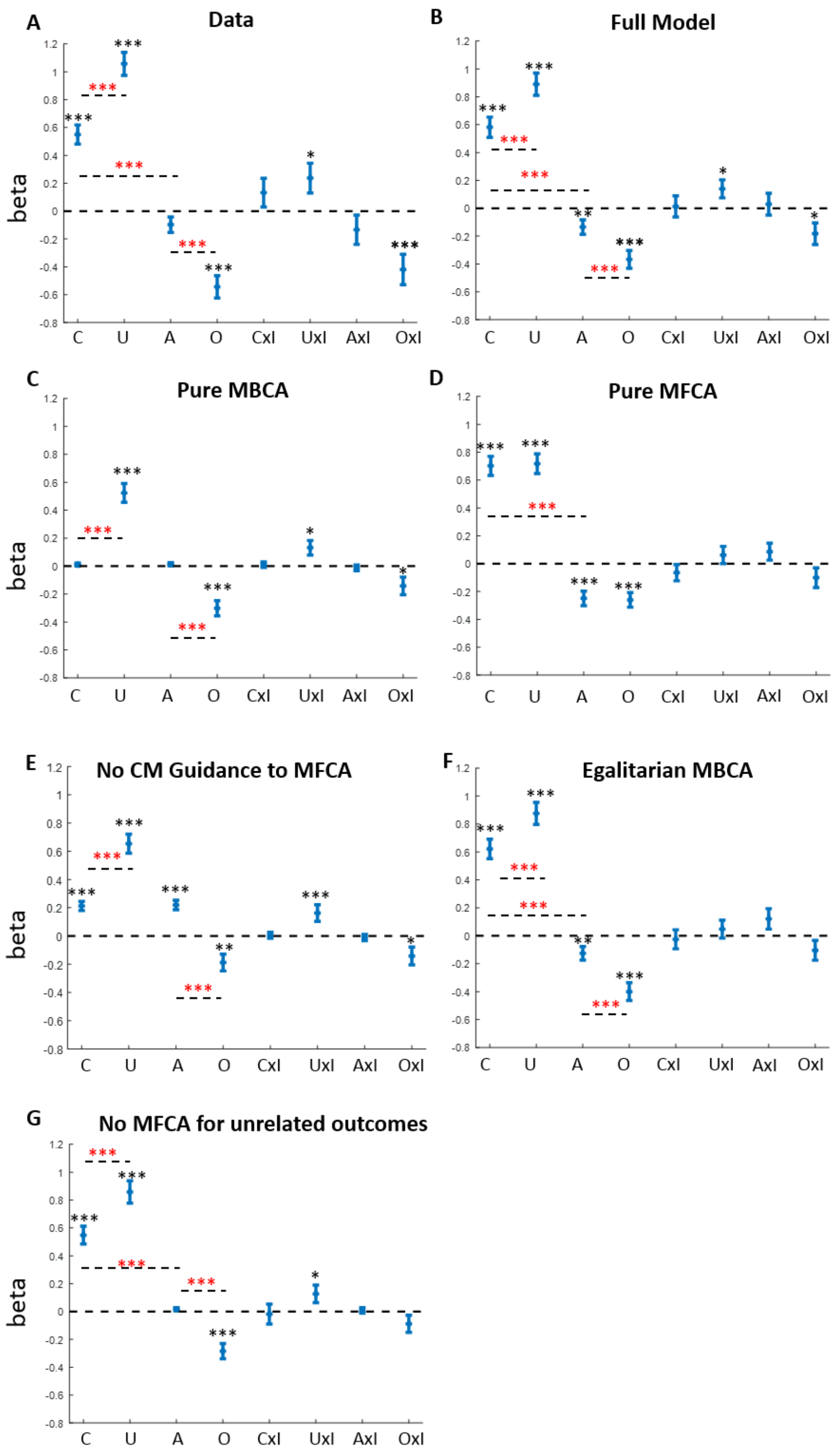

Figure S2, related to Fig. 2. Signatures of MBCA and MFCA. A) Fig. 2B (empirical effects on choice repetition) copied for convenience. B) Corresponding panel for our full model, which captured all effects of interest. C) Same but for the pure MBCA model, which failed to capture the Common effect on choice repetition, and the difference between the Common and Absent effects— Signature of MFCA and of MB guidance to

MFCA, respectively. D) Same but for the pure MFCA model, which failed to capture the contrast effects of Common vs. Unique and Absence vs. Other— signatures of MBCA. Additionally, the model failed to predict the Importance interactions with Unique and Counterfactual— Signatures of importance based MBCA. E) Same but for the ‘No CM guidance for MFCA model’, which failed to predict a difference between the Common and Absent effects— a signature of MB guidance for MFCA. F) Same but for the ‘egalitarian MBCA’ model, which failed to predict to interaction effects for Unique and Other outcomes with Importance— signatures of importance based MBCA. G) Same but for the ‘no MFCA for choice-unrelated outcomes’ sub-model. Unlike the full model, this model cannot predict a negative effect for the Absent outcome and failed to predict the interaction between Other and Importance. Error bars correspond to SEM across participants calculated separately in each condition (n=42). *,**,*** denote p<.05, .01, .001, respectively. p-values were calculated based on mixed effects logistic regression models.

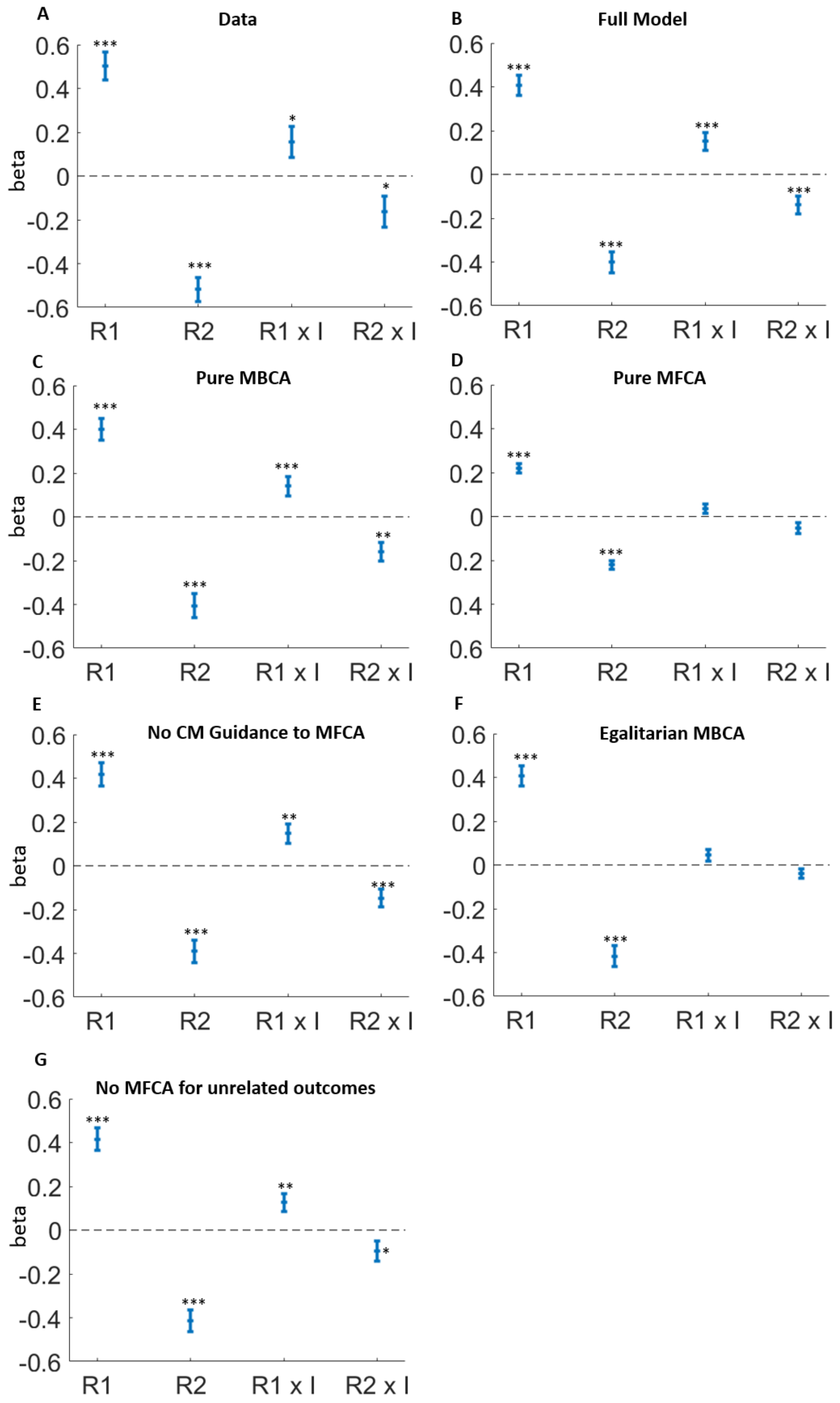

Figure S3. A signature of importance based modulation of MBCA based on all trial-to trial transitions. A) On each trial (n+1) the two offered individuals grow one vegetable in common, and each grows one unique vegetable. We used a mixed effects logistic model (Methods) to regress the probability to choose the person presented on the right side of the display as a function of whether his/her unique vegetable was previously (trial n)

rewarded (denoted R1), whether the other (left-side) person's unique vegetable was previously rewarded (denoted R2), and whether these two vegetables were important on the previous trials (denoted I). We found that a previous reward to each person's unique vegetable increased the probability to choose that person and that critically, these effects were stronger when the 2 unique vegetables were important on the previous trial, as evident from the interactions between rewards and importance. B) Corresponding panel for our full model, which captured all effects of interest. Similarly, all other models that allowed for importance based modulation of MBCA, accounted for these effects (C, E, G). In contrast, while the pure MFCA (in which MBCA was altogether absent; D) and the egalitarian MBCA (in which MBCA was not modulated by importance; F) accounted for the reward effects, they did not account for the interactions between reward-outcomes and importance. We thus interpret these interactions as a signature of importance based modulation of MBCA. The arrangement of this figure is similar to Fig. S1.

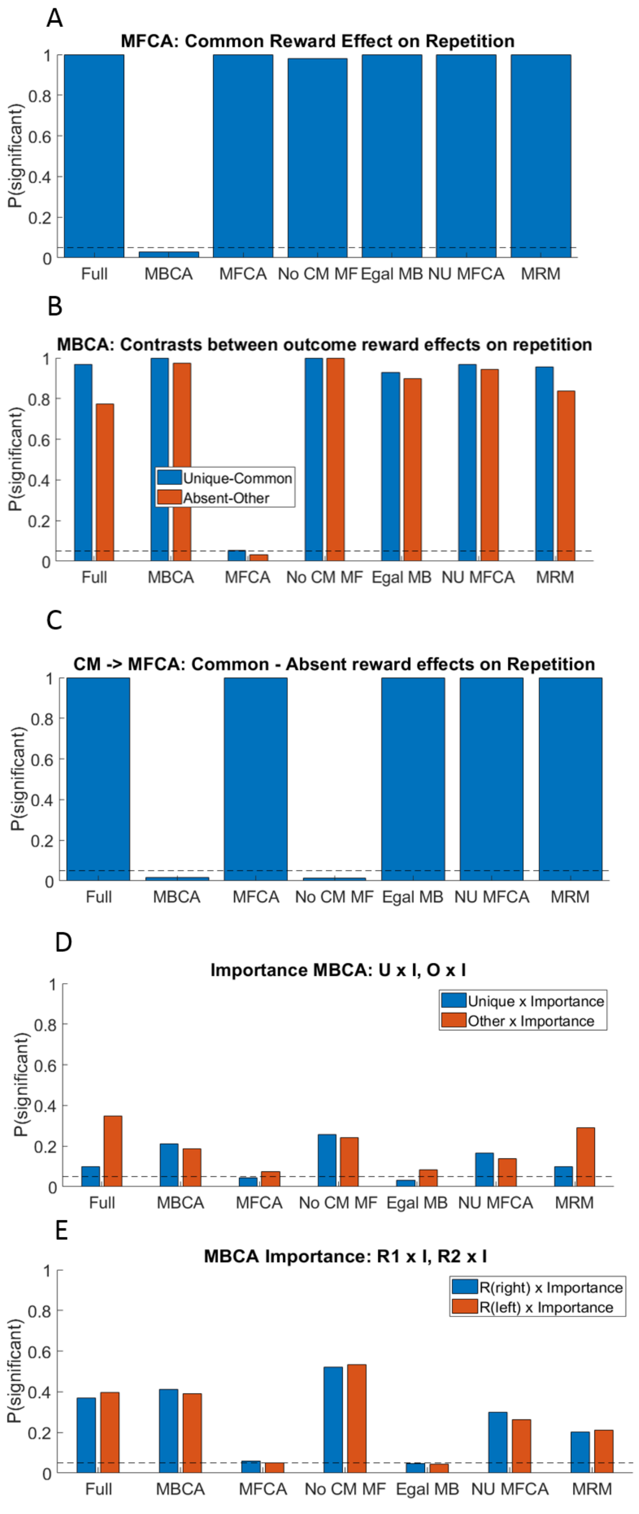

Fig. S4. Related to Fig. 2. Signatures of MBCA and MFCA. In Figs. S2-S3 we presented model-simulation results based on data sets that were larger than the actual empirical data (for each participants we created 25 data sets). While this method mitigates noise in the calculation of model predictions it doesn't show how likely model-agnostic signatures of interest are to emerge from the various models for data of the same size as the empirical

data. To address this question, for each sub model, we simulated 1 data set per participant and ran our “model-agnostic” mixed-effects models as for the real data. We repeated this procedure 1001 times and calculated the proportion of simulations which yielded a significant ($p<.05$) model-agnostic effect of interest (y axes). A) Positive MFCA for a related outcome (blue; Common outcome effect corresponding to Fig. 2B). This effect is detected in a high proportion of simulations for all models except the pure MBCA sub-model which lacks a MFCA component. B) The two MBCA signatures (blue: Unique-Common outcome effect corresponding to Fig. 2B; orange- Absent-Other outcome effect corresponding to Fig. 2B). Both signatures are detected in a high proportion of simulations for all models except for the pure-MFCA sub-model which lacks a MBCA component. C) CM guidance to MFCA signature (corresponding to the Common – Absent contrast in Fig. 2B). The effect is almost always detected for all models except for the pure MBCA (which lacks a MFCA component) and the No CM guidance to MFCA (which does not allow for such guidance), sub-models. D) Importance modulation on MBCA signatures (blue: Unique x Importance effect; orange: Other x Importance effect corresponding to Fig. 2B). The first (U x I) effect is detected in more than 5% of the simulations for all models except for the pure MFCA (which excludes MBCA) and the egalitarian MBCA (which doesn’t allow for importance based modulation) sun-models. The second (O x I) effect is also detected less frequency in these two sub-models but it is detected with probability ~.08 (SE=.007). E) Our second set of signatures of importance based modulation on MBCA based on all trial (blue: Right-reward x Importance effect; orange: Left-reward x Importance effect corresponding to Fig. 2C) provide a better discrimination between the models that allow for such modulation and the two sub-models that do not. x-axes show the model: *‘Full’: Full model. MBCA/MFCA: pure MBCA.MFCA sub-models; No CM MF:* no CM guidance for MFCA sub-model; Egal MB: Egalitarian MBCA sub-model; NU MFCA- No MFCA for unrelated choice outcomes sub-model; MRM- “most responsible model”- each participant was simulation using the modes that had the highest posterior probability to account for their data. The dashed black lines correspond to detecting effects in 5% of the simulations.

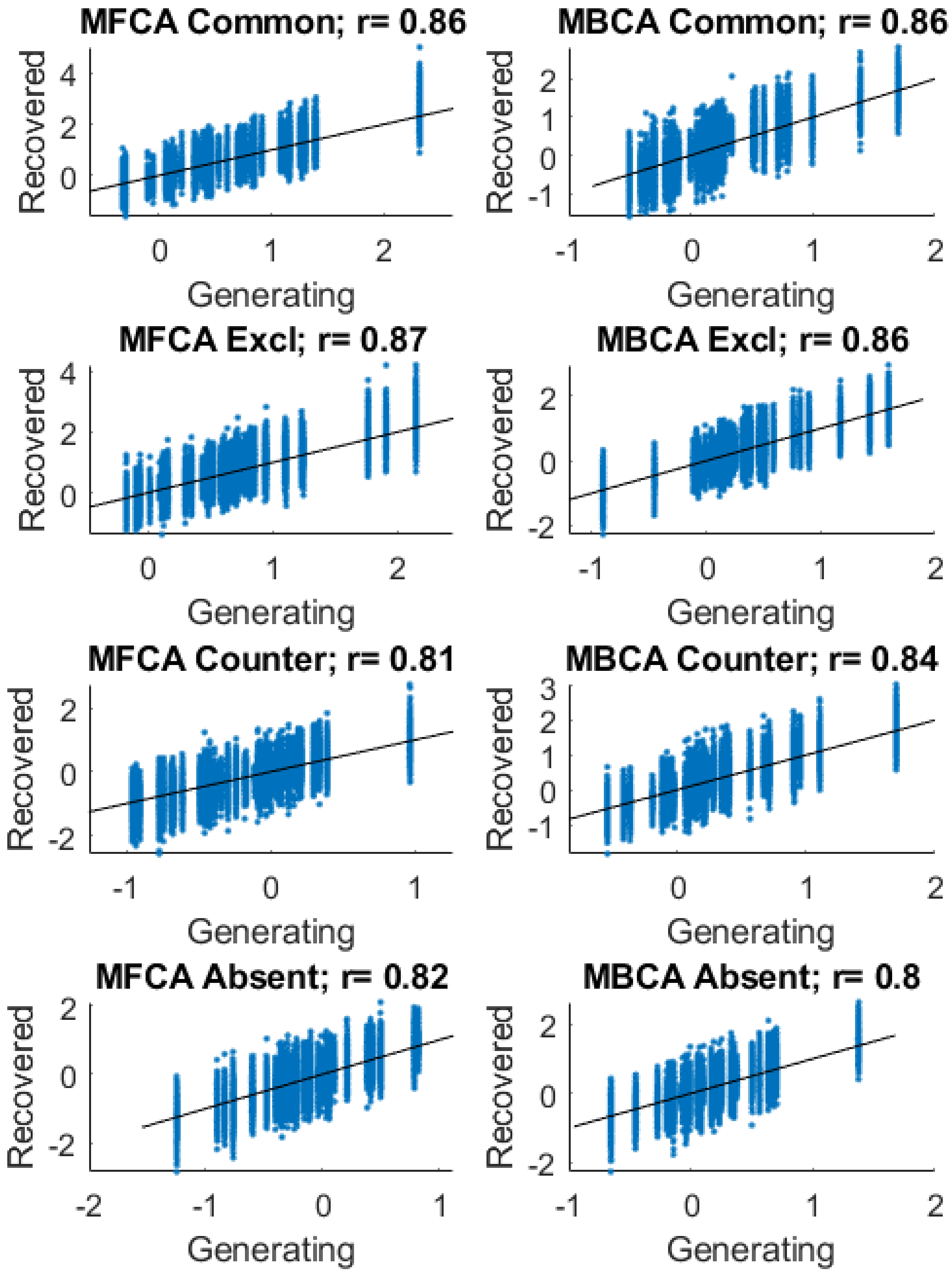

Fig. S5. Parameter recovery for the full model at the level of individual participants. Each panel displays a scatter plot of a generating CA parameter (abscissa) vs. the recovered parameters (ordinate; SI Methods). Black solid lines are imposed diagonals. Model recovery for all CA parameters was high [All Pearson's r >= .8].

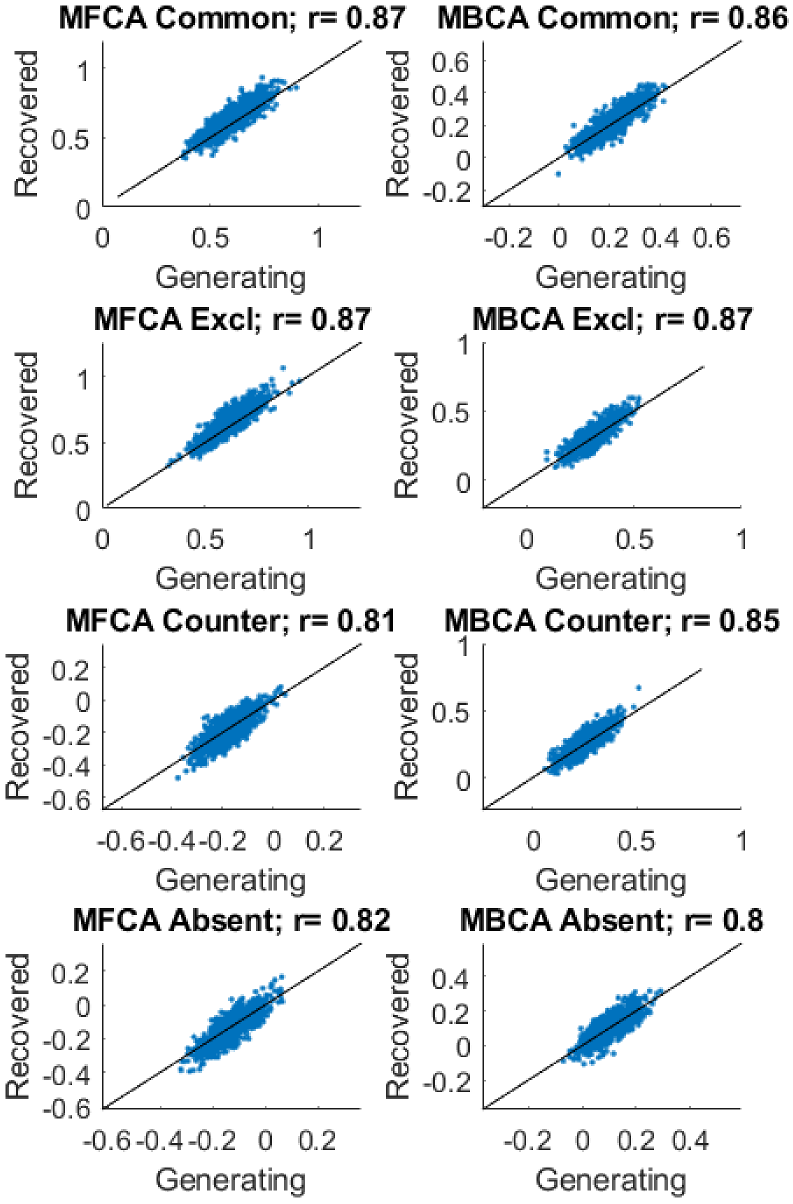

Fig. S6. Parameter recovery for full model group-averaged parameters. Same as Fig. S3 but here group averages across 42 bootstrapped participants are plotted, rather than single-subject parameters (SI Methods).

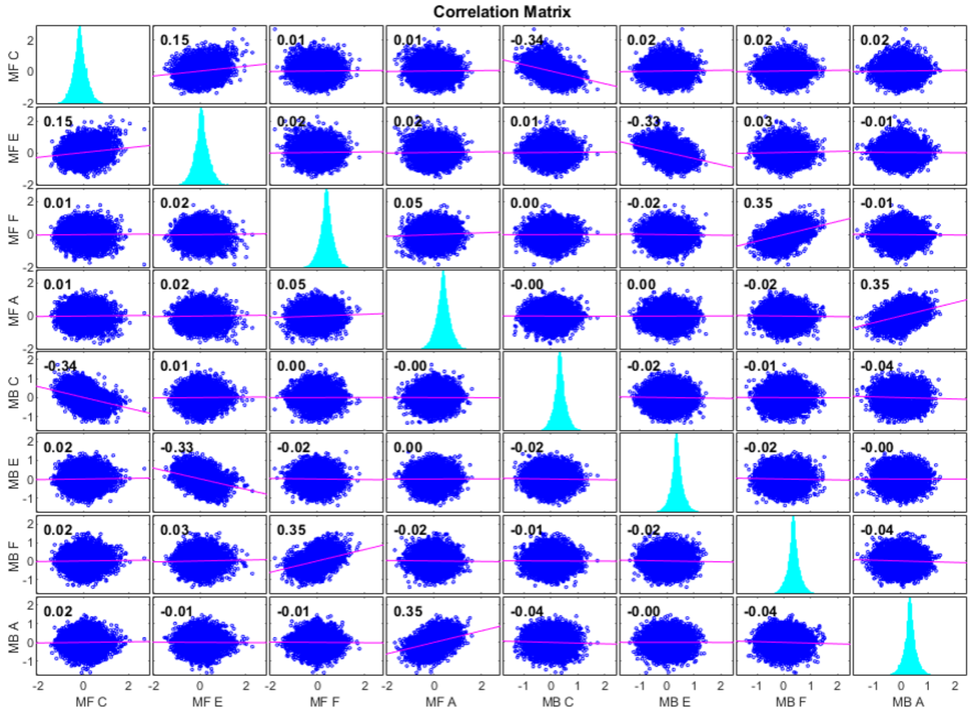

Figure S7. Parameter trade-offs in the full model at the level of individual participants. Each non-diagonal panel displays a scatter plot between estimation errors (recovered minus generated parameters) for a pair of CA parameters (C-common, E-exclusive, F-counterfactual, A-absent). The numbers in the top left are Pearson's r and regression lines are imposed. Diagonal panels display estimation-error distributions for the various CA parameters. The figure shows there are modest pairwise trade-offs between MBCA and MFCA parameters corresponding to the same outcome type (e.g., MB C and MF C; abs(r)~ .35). For other pairs of CA parameters trade-offs are negligible. We also assessed whether these trade-offs can affects the independence of our two effects of main interest involving relatedness and importance (Fig. S5).

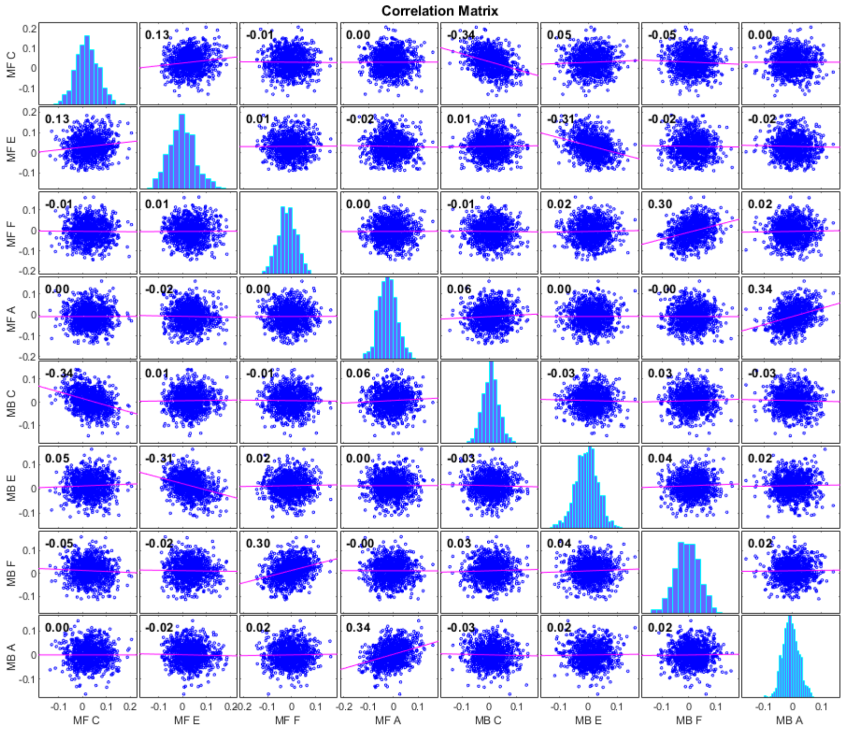

Fig. S8. Trade-offs in the full model for group-averaged parameters. Same as Fig. S3 but here group averages across 42 bootstrapped participants are plotted, rather than single-subject parameters (SI Methods).

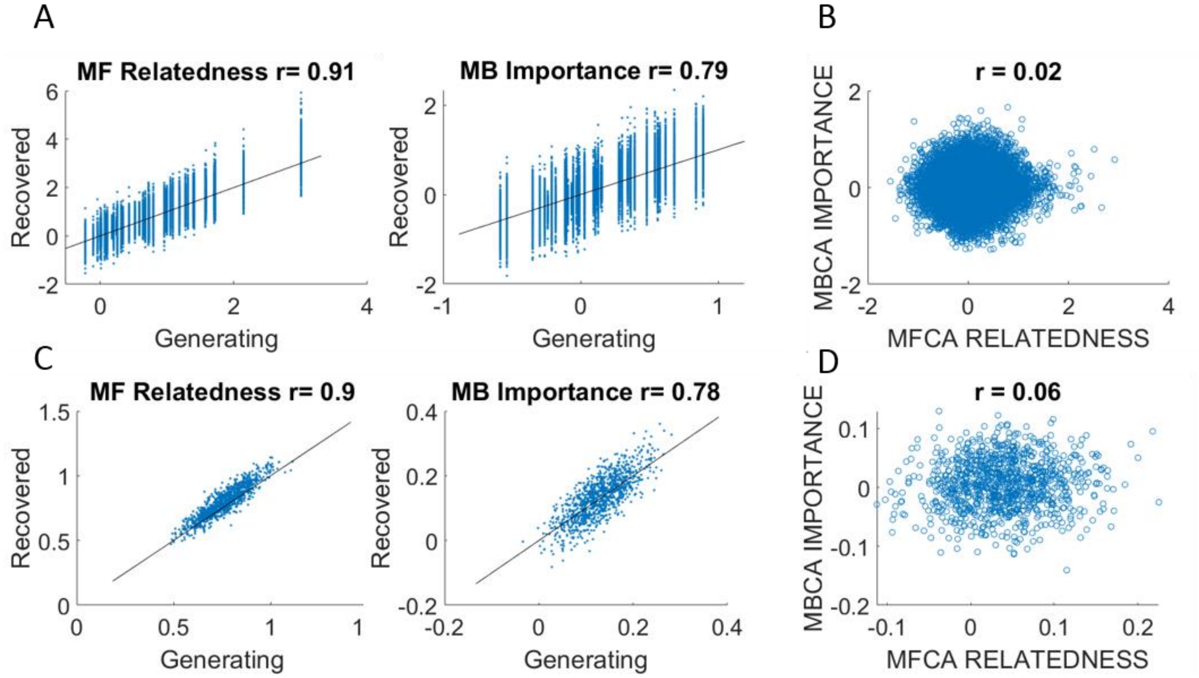

Figure S9. Recovery and trade-off for the effects of main interest. A) The left panel presents the full model's recovery of the relatedness effect on MFCA for individual participants. Generating effects (abscissa) are scattered vs. recovered effects (SI Methods). A diagonal line is imposed. The right panel is the same but for the importance effect on MBCA. Both effects are recovered with good fidelity. B) A scatter plot of estimation errors for the two effects shows that they are negligibly correlated (r=.02). Thus, the interpretations of these effects are not confounded by parameter trade-offs. C-D) Same as A, B but for group averages across 42 bootstrapped participants, rather than single-subject parameters (SI Methods).

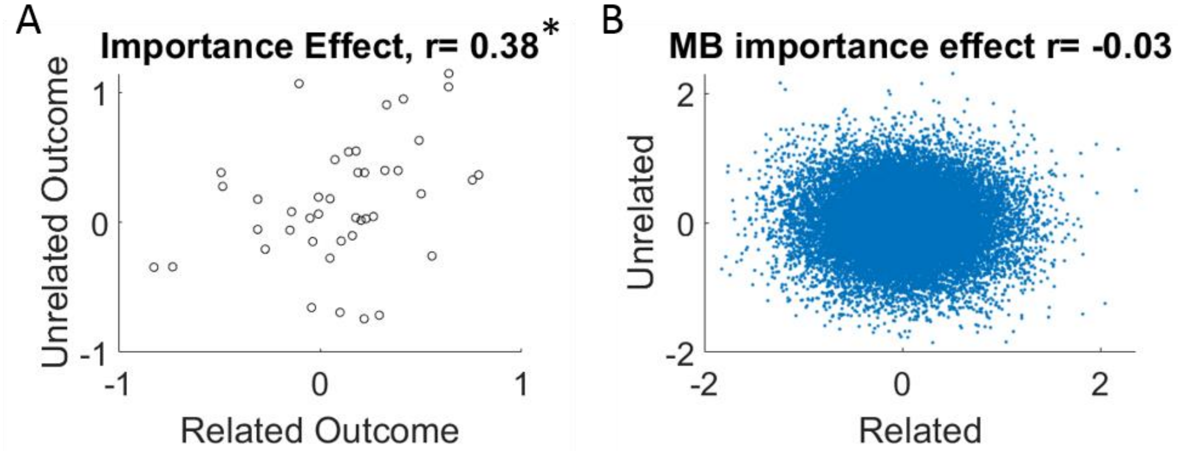

Fig S10. Importance based modulation of MBCA as a function of choice relatedness. A) We calculated, based on the ML full-model parameters, importance-based modulation of MBCA effects separately for the choice related (exclusive – common MBCA parameters) and unrelated (counterfactual – absent MBCA parameters) outcomes. These two effects correlated positively (r= .38, p=.013). B) This positive correlation was not due to parameter trade-offs as our recovery simulations (SI Methods) showed a negligible correlation between estimation-errors (plotted) for these two effects.

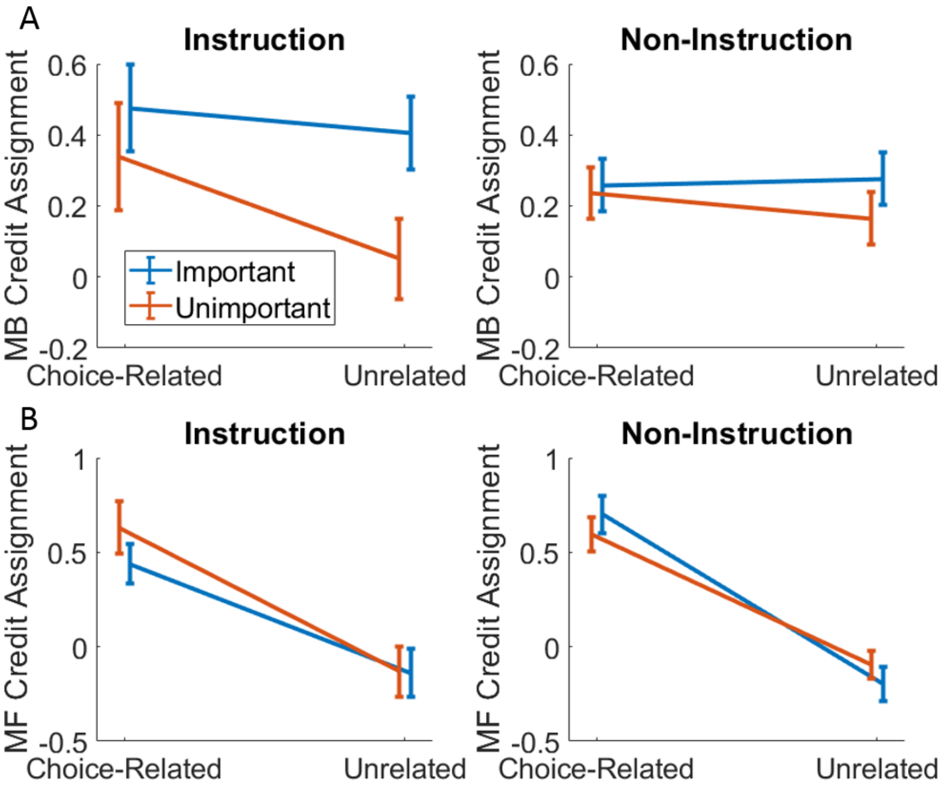

Fig S11. Related to Fig. 4. Experimental blocks 1 and 4 included transition structure reminders (Methods). To examine whether these reminders affected CA patterns, we fitted our full model separately to blocks that included, or did not include, reminders. We then repeated our mixed effects models, adding as a third regressor (in addition to relatedness and importance) a transition structure reminder indicator (Methods). None of the effects involving this regressor was significant (MBCA: all $p>.179$; MFCA: all $p>.196$), and these mixed effects models supported the very same conclusions as those that omitted this regressor (reported in the main text). We note that cognitive map guidance to MFCA, as well as MBCA and its modulation by importance, all rely exquisitely on knowledge of the transition structure. Thus, had the transition structure been subject to substantial forgetting, we would have expected both a positive Instruction effect as well as a positive importance x instruction interaction effect on MBCA, and a positive Relatedness x Instruction interaction effect on MFCA. As none of these effect was significant, there is no evidence that participants forgot the transition structure on non-instruction blocks.

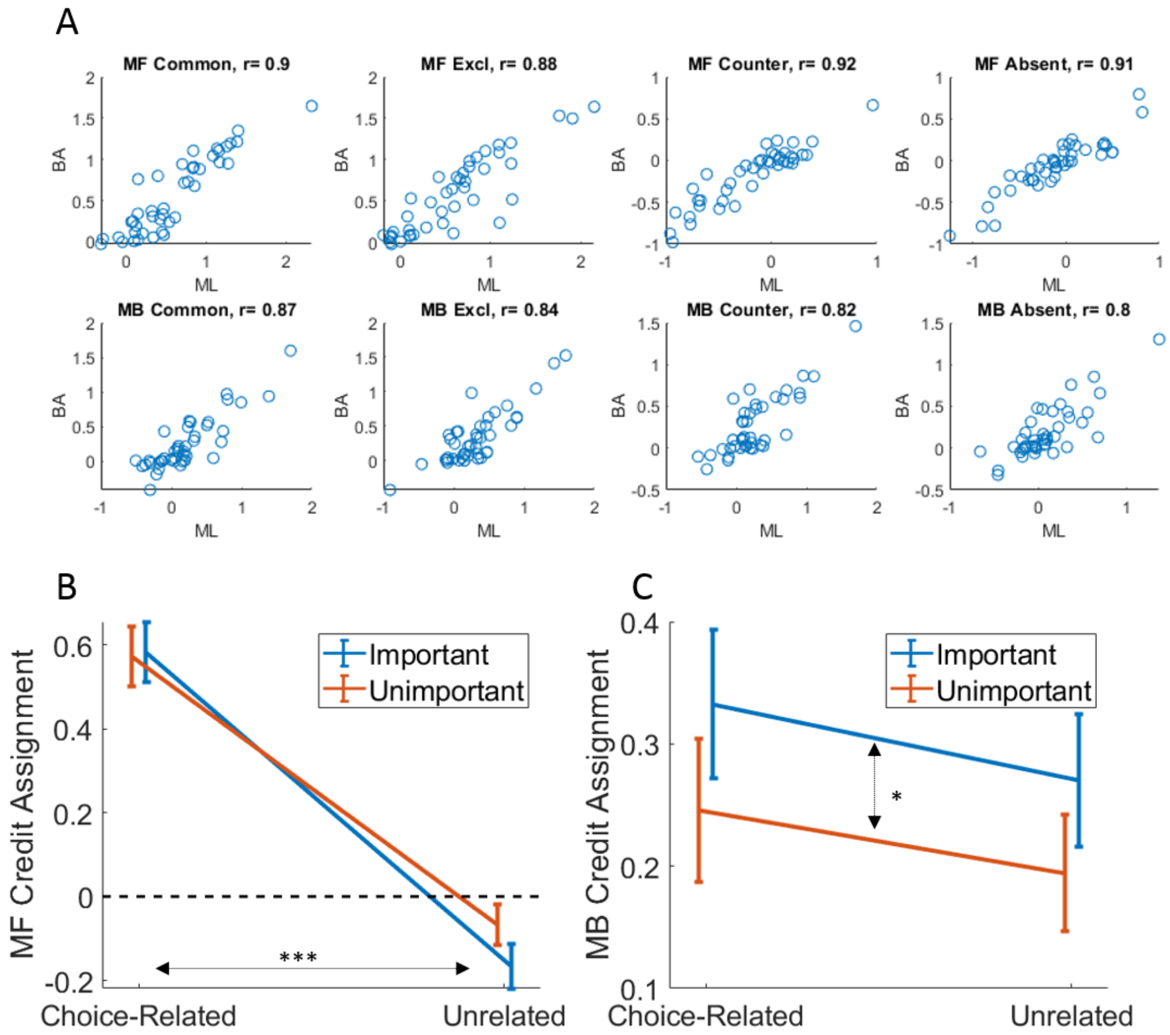

Fig S12. Related to Fig. 4. Analysis of Parameters based on Bayesian Averaging across models. A) Scatter plots for CA parameters obtained based on ML-estimation for the full model (x-axes) or by Bayesian averaging (y-axes). Corresponding parameters correlated positively and strongly (all r> .8) across participants. B-C) Same as Fig. 4B-C but for the BA (rather than ML) parameters. Mixed effects models (same as for Fig. 4) supported the same conclusions about CA as those based on ML parameters. For MFCA We found a positive main effect for choice 'relatedness' (b= 0.69, t(164)= 7.76, p= 8e-13). Neither the main effect for 'nor the interaction between 'importance' and 'relatedness' were significant (both p> .11). Additionally, whereas MF credit assignment for choice-related vegetable was positive (b=0.58, F(1,164)= 75.96, p= 3e-15), it was negative for choice-unrelated vegetables (b=-0.12, F(1,164)= 7.85, p= .006). For MBCA we found a positive main effect for 'importance' (b= 0.08, t(164)= 2.52, p= .013). Neither the main effect for choice-'relatedness' nor the interaction between 'importance' and 'relatedness' were significant (both p>.203). Furthermore, MBCA was positive for both 'important' (b= 0.3, F(1,164)= 33.92, p= 3e-8) and 'unimportant' (b= 0.22, F(1,164)= 24.52, p= 2e-6) outcomes.

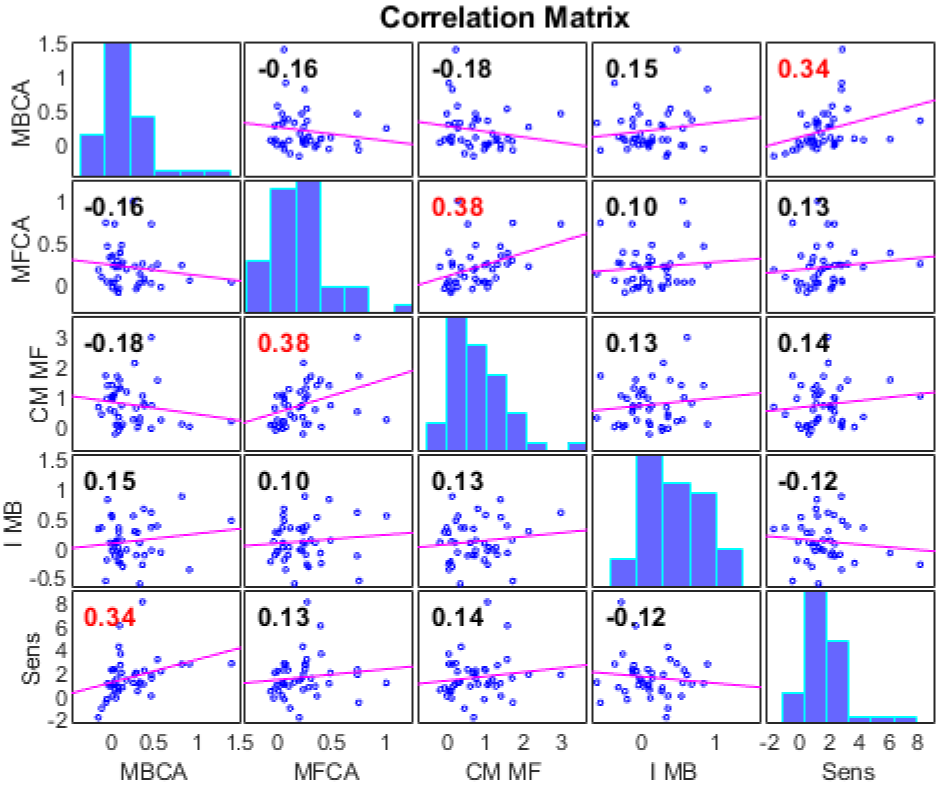

Fig. S13. Correlation between different measures of CA. The figure presents across-participants correlations between each pair of variables in the following set: Overall levels of MBCA (an average of all 4 MBCA parameters; denoted MBCA), overall level of MFCA (an average of all 4 MFCA parameters; denoted MFCA), CM guidance to MFCA (the contrast between average MFCA parameters for related and unrelated outcomes; denoted CM MF), and importance-based modulation of MBCA (the contrast between average MBCA parameters for important and unimportant outcome; denoted I MB) and our model-agnostic measure of choice-sensitivity (denoted Sens). Red: Significant correlations ($p<.05$). Diagonal panels display distributions for the various measures.

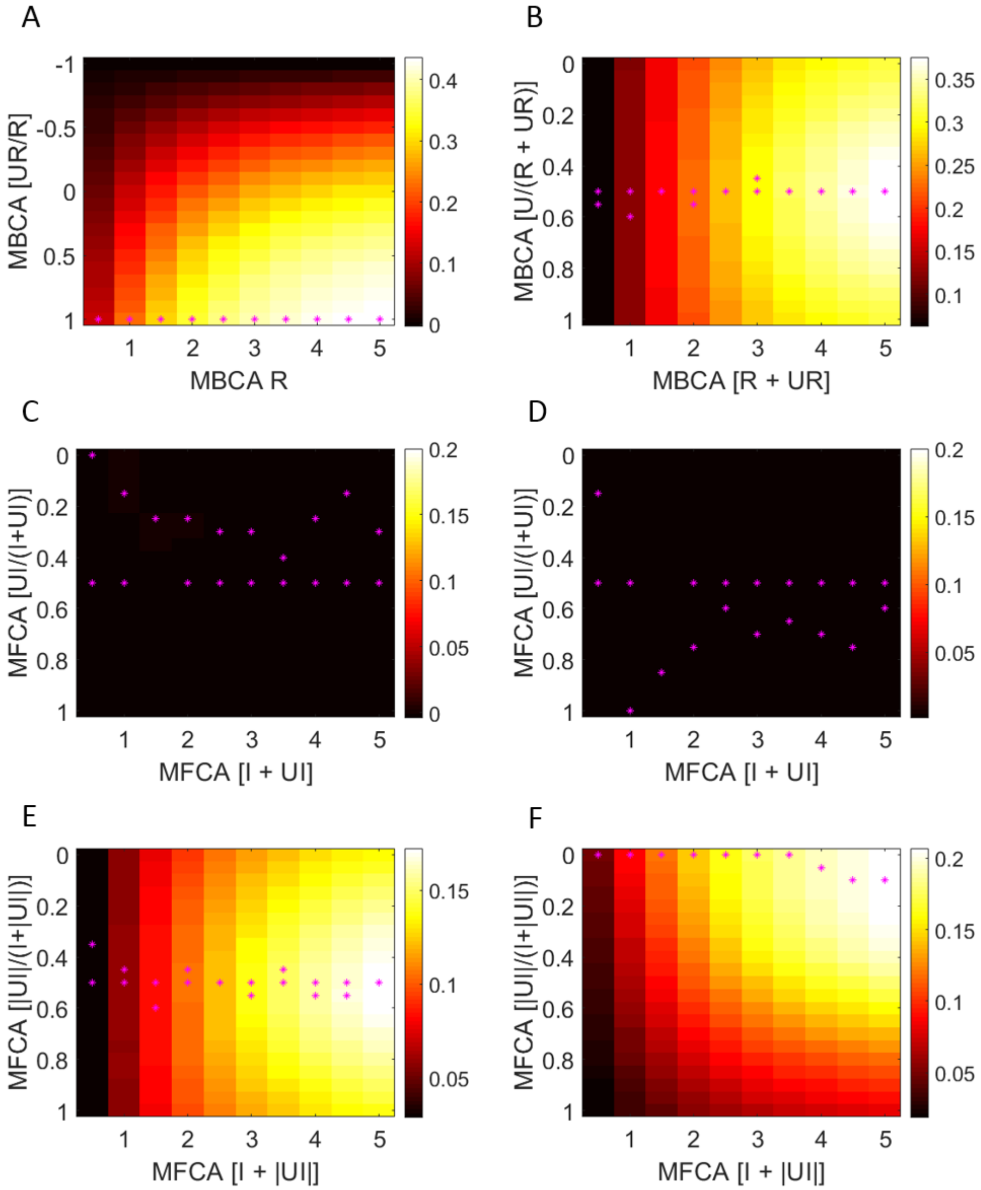

Fig S14. Related to Fig. 5. *Simulated reward earnings for various regimes of CA parameters. A) Same as Fig. 5A but for pure MB agents. Earnings are maximised when the ratio is 1 i.e., when MBCA for unrelated outcome is equal to MBCA for related outcomes. B) Standardized earning for pure-MB agents as a function of total MBCA for related and unrelated outcomes (abscissa) and the proportion of unrelated MBCA (ordinate).Here, MBCA is positive for both related and unrelated outcomes. Earnings are maximised when the proportion of MBCA for unrelated outcome is .5 i.e., when related and unrelated MBCA are equal. Taken together, A-B show that MBCA is optimal when MBCA for related and unrelated outcomes is equal. C) Same as Fig. 5C but for pure MFCA. D) Same as C but for a modified variant of the task wherein outcome-importance is positively correlated across trials. In both C and D, earnings are very close to random for all parameter combinations. Thus, MFCA cannot lead to substantial reward*

acquisition by merely assigning more credit to important than unimportant outcomes. This is expected as the MFCA regime simulated here does not take into account choice-relatedness. E) Same as C, but here, MFCA for unrelated outcomes is negative and equal in magnitude to MFCA for related outcomes. Standardized earnings are maximal when the magnitude of MFCA is equal for important and non-important outcomes. F) Same as E but for a modified variant of the task. MFCA benefits from importance-based modulation, that is, when CA is enhanced for important outcomes. Magenta asterisks mark the maximal earning in each column. However, if the maximal earnings was not significantly higher than earnings in the central row of that column, we also marked the central row (hence some columns feature 2 asterisks).

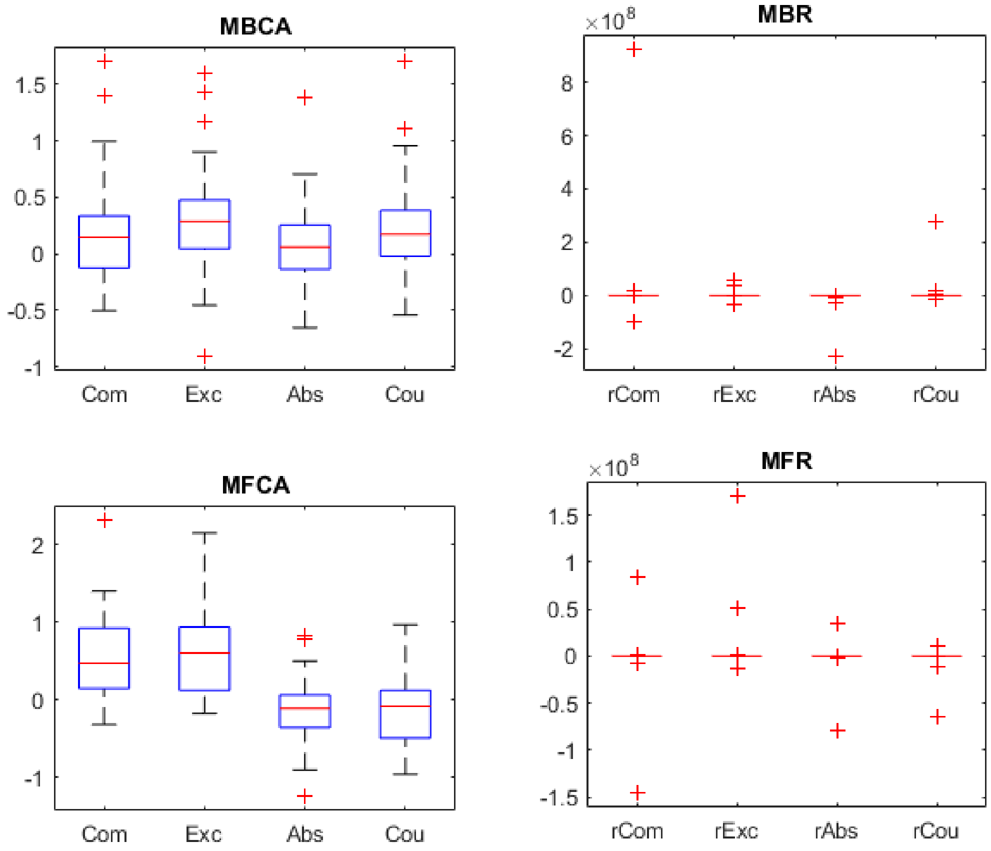

Fig. S15. Box plots for MB (top panels) and MF (bottom panels) "ca" (left panels) and "r" (right panels; See "Modelling Choices" note) parameters for the different outcome types (**Com**mon, **Exc**lusive, **Abs**ent, **Cou**nterfactuals). The "ca" parameters provide for a more stable parametrisation, whereas the "r" parametrisation leads to what are numerically huge values (one cannot even see the boxes). Box plots were created using Matlb's routing "boxplot" with default settings.

Param	c^{MF}_{shared}	$c^{MF}_{exclusi}$	$c^{MF}_{counter}$	c^{MF}_{missed}	c^{MB}_{shared}	$c^{MB}_{exclusi}$	$c^{MB}_{counter}$	c^{MB}_{missed}	f^{MF}	f^{MB}	f^{P}	pr
Mean (SE)	0.60 (0.08)	0.62 (0.09)	-0.18 (0.07)	-0.12 (0.07)	0.21 (0.07)	0.31 (0.07)	0.25 (0.07)	0.1 (0.07)	.5 (.06)	.38 (.06)	.54 (.06)	-0.21 (0.07)

Table S1. Best fitting parameters for the full model (See 'Computational Models' in methods for a full description of the model and its parameters).

SI References

1. Moran R, Goshen-Gottstein Y. Old processes, new perspectives: Familiarity is correlated with (not independent of) recollection and is more (not equally) variable for targets than for lures. *Cogn Psychol*. 2015;79:40-67. doi:10.1016/j.cogpsych.2015.01.005
2. Chopin N. A sequential particle filter method for static models. *Biometrika*. 2002;89(3):539-552. doi:10.1093/BIOMET/89.3.539
3. Findling C, Skvortsova V, Dromnelle R, Palminteri S, Wyart V. Computational noise in reward-guided learning drives behavioral variability in volatile environments. *Nat Neurosci*. 2019;22(12):2066-2077. doi:10.1038/s41593-019-0518-9
4. Stephan KE, Penny WD, Daunizeau J, Moran RJ, Friston KJ. Bayesian model selection for group studies. *Neuroimage*. 2009;46(4):1004-1017. doi:10.1016/j.neuroimage.2009.03.025
5. Rigoux L, Stephan KE, Friston KJ, Daunizeau J. Bayesian model selection for group studies - Revisited. *Neuroimage*. 2014;84:971-985. doi:10.1016/j.neuroimage.2013.08.065
6. Moran R. Thou shalt identify! The identifiability of two high-threshold models in confidence-rating recognition (and super-recognition) paradigms. *J Math Psychol*. 2016;73:1-11. doi:10.1016/j.jmp.2016.03.002
